# Comprehensive Analysis of Alteration Landscape and Its Clinical Significance of Mitochondrial Energy Metabolism Pathway-Related Genes in Lung Cancers

**DOI:** 10.1155/2021/9259297

**Published:** 2021-12-20

**Authors:** Zhen Ye, Huanhuan Zhang, Fanhua Kong, Jing Lan, Shuying Yi, Wenshuang Jia, Shu Zheng, Yuna Guo, Xianquan Zhan

**Affiliations:** ^1^Department of Thoracic Surgery, Shandong Provincial Hospital Affiliated to Shandong First Medical University, 324 Jingwu Weiqi Road, Jinan, Shandong 250021, China; ^2^Department of Biochemistry and Molecular Biology, School of Basic Medicine, Shandong First Medical University, 6699 Qingdao Road, Jinan, Shandong 250117, China; ^3^Medical Science and Technology Innovation Center, Shandong First Medical University, 6699 Qingdao Road, Jinan, Shandong 250117, China; ^4^Department of Thoracic Surgery, Tai'an City Center Hospital, Tai'an, Shandong 271000, China; ^5^Shandong Key Laboratory of Radiation Oncology, Shandong Cancer Hospital and Institute, Shandong First Medical University, 440 Jiyan Road, Jinan, Shandong 250117, China

## Abstract

**Background:**

Mitochondria are the energy factories of cells. The abnormality of mitochondrial energy metabolism pathways is closely related to the occurrence and development of lung cancer. The abnormal genes in mitochondrial energy metabolism pathways might be the novel targets and biomarkers to diagnose and treat lung cancers.

**Method:**

Genes in major mitochondrial energy metabolism pathways were obtained from the KEGG database. The transcriptomic, mutation, and clinical data of lung cancers were obtained from The Cancer Genome Atlas (TCGA) database. Genes and clinical biomarkers were mined that affected lung cancer survival. Gene enrichment analysis was performed with ClusterProfiler and the gene set enrichment analysis (GSEA). STRING database and Cytoscape were used for protein-protein interaction (PPI) analysis. The diagnostic biomarker pattern of lung cancer was optimized, and its accuracy was verified with 10-fold cross-validation. The four genes screened by logistic regression model were verified by western blot in 5 pairs of lung cancer specimens collected in hospital.

**Results:**

In total, 188 mitochondrial energy metabolism pathway-related genes (MMRGs) were included in this study. GSEA analysis found that MMRGs in the lung cancer group were mainly enriched in the metabolic pathway of oxidative phosphorylation and electron respiratory transport chain compared to the control group. Age did not affect the mutation frequency of MMRGs. Comparative analysis of these 188 MMRGs identified 43 differentially expressed MMRGs (24 upregulated and 19 downregulated) in the lung cancer group compared to the control group. The survival analysis of these 43 differentially expressed MMRGs found that the survival time was better in the low-expressed GAPDHS group than that in the high-expressed GAPDHS group of lung cancers. The advanced age, high expression of GAPDHS, low expressions of ACSBG1 and CYP4A11, and ACOX3 mutation were biomarkers of poor prognosis in lung cancers. PPI analysis showed that proteins such as GAPDH and GAPDHS interacted with many proteins in mitochondrial metabolic pathways. A four-MMRG-signature model (*y* = 0.0069∗ACADL − 0.001∗ALDH18A1 − 0.0405∗CPT1B + 0.0008∗PPARG − 1.625) was established to diagnose lung cancer with the accuracy up to 98.74%, AUC value up to 0.992, and a missed diagnosis rate of only 0.6%. Western blotting showed that ALDH18A1 and CPT1B proteins were significantly overexpressed in the lung cancer group (*p* < 0.05), and ACADL and PPARG proteins were slightly underexpressed in the lung cancer group (*p* < 0.05), which were consistent with the results of their corresponding mRNA expressions.

**Conclusion:**

Mitochondrial energy metabolism pathway alterations are the important hallmarks of lung cancer. Age did not increase the risk of MMRG mutation. High expression of GAPDHS, low expression of ACSBG1, low expression of CYP4A11, mutated ACOX3, and old age predict a poor prognosis of lung cancer. Four differentially expressed MMRGs (ACADL, ALDH18A1, CPT1B, and PPARG) established a logistic regression model, which could effectively diagnose lung cancer. At the protein level, ALDH18A1 and CPT1B were significantly upregulated, and ACADL and PPARG were slightly underexpressed, in the lung cancer group compared to the control group, which were consistent with the results of their corresponding mRNA expressions.

## 1. Introduction

Lung cancer has become an important public health problem due to its high morbidity and mortality. Metabolic changes in lung cancer affect prognosis and response to treatment [1]. Mitochondrial translation defects are the basis of many inborn errors of metabolism, which has been linked to multiple diseases such as cancer [2]. Mitochondrial pathway abnormalities and metabolic disorders can lead to gene expression changes to promote cancer development, progression, and immune system evasion [3]. Monoubiquitination of histone H2B negatively regulates the Warburg effect and tumorigenesis of human lung cancer cells through controlling the expressions of multiple mitochondrial aspiration genes [4]. Functional changes of mitochondrial proteins have great influence on the development and progression of lung cancer [5]. Studies have shown that apoptosis-inducing factor regulates mitochondrial respiration, and phosphorylation promotes the progression of lung cancer [6]. Mitochondrial ribosome-related genes were identified as the highest expressed genes to associate with metastasis-specific lethality in lung adenocarcinoma. Pyruvate carboxylase (PC) is a mitochondrial enzyme and is associated with lung metastasis in breast cancer [7]. Tumor cells rely on glycolysis and mitochondrial oxidative phosphorylation to survive. The mitochondrial oxidative phosphorylation pathway has become an increasingly interesting area of cancer therapy [8]. The metastatic cell state of lung adenocarcinoma is related to the specific changes of mitochondrial function, which opens up a new way for the specific treatment of metastatic lung adenocarcinoma [9]. Lung cancer shows a strong mitochondrial glucose oxidation. Mitochondrial electron transport chain (MTC) is essential for tumor growth. Inhibition of MTC has been proved to have antitumor effect in combination with targeted therapy [10]. VDAC1, a mitochondrial protein that controls cellular energy, is often overexpressed in many cancers. By specifically silencing VDAC1 gene, the growth of lung cancer cells can be inhibited [11]. Therefore, mitochondrial energy metabolism pathways not only affect the occurrence and development of lung cancer but also are a potential target of lung cancer therapy.

Metabolism in living organisms includes anabolism and catabolism. The main locations of anabolism are in the cytoplasm of cells, such as cholesterol synthesis, fatty acid synthesis, and glycogen synthesis. The main locations of catabolism are in mitochondria, such as the Kreb's cycle of the three nutrients, the *β* oxidation of fatty acids, and the aerobic oxidation of glucose. Many substances are metabolized in the cell's mitochondria. Common and important metabolisms are oxidative decomposition of glucose, tricarboxylic acid cycle, and oxidative phosphorylation, etc. This study focuses on mitochondrial energy metabolism pathway, especially the major catabolic pathways in mitochondria. One important goal of mitochondrial catabolism is to convert the chemical energy stored in the three main nutrients into energy that the body can use, such as heat and ATP. Thus, the expression and mutation abnormalities of mitochondrial energy metabolism pathway-related genes (MMRGs) could cause an energy production abnormality, which are associated with the occurrence and development of malignant tumors. For example, GAPDHS, an important gene in the glycolytic pathway, is highly expressed in melanoma and is a biomarker of poor prognosis [12]. ACSBG1, an acyl-CoA synthetase, is originally identified in the fruit fly mutant bubblegum. Acyl-CoA synthase activates fatty acids to produce its coenzyme A derivatives and plays an important role in fatty acid metabolism [13]. ACSBG1 is involved in the metabolic pathway of fatty acids, which is significantly downregulated in psoriatic lesions [14]. Cytochrome P4504 (CYP4) family of enzymes is involved in the metabolisms of fatty acids and signaling molecules, including eicosanes, leukotrienes, and prostatinoids. CYP4 enzyme participates in maintenance of the normal range of fatty acids and fatty acid-derived bioactive molecules [15], such as CYP4A11 is downregulated in liver cancer tissues [16].

Mitochondrial energy metabolism pathway abnormalities are also associated with oxidative stress that is derived from various reasons, including production of too many free radicals, releasing of a large number of reactive oxygen species (ROS), and the body's own ability to resist oxidation, which causes the accumulation of excessive ROS, and imbalance of oxidation and antioxidation abilities. Oxidative stress is a hallmark of many cancers. Generally, cancer cells have higher levels of ROS than normal cells [17, 18]. Mitochondrial respiration is a major source of ROS, and the high-level ROS can damage organelles [19]. The electron leakage of electron transport chain complexes I and III located in the mitochondrial inner membrane leads to partial oxygen reduction to form superoxides, which spontaneously mutates to form hydrogen peroxide [20]. Mitochondrial DNA is vulnerable to damage caused by somatic mutations, which causes dysfunction of mitochondrial respiratory chain and energy production to promote ROS production and enhance tumorigenicity [21]. Cancer cells are highly metabolically active and hypoxic. Due to continuous growth and inadequate vascular perfusion, cancer cells are prone to increase ROS that can diffuse through mitochondrial membrane and destroy DNAs [22]. Mitochondria play an important role in the occurrence, development, and metastasis of cancers, which might be therapeutic targets of drugs [23]. In addition, the redox signaling produced by mitochondrial ROS is related to the occurrence and development of cancer [24]. Targeted nicotinamide adenine dinucleotide metabolism has emerged as a potential treatment to improve age-related diseases and extend human healthy life spans [25]. The acid-activated mitochondria-targeting and redox-responsive nanomicelles could be a new hope for cancer treatment. Some studies found that PDIC-NC might target mitochondria as a respiratory inhibitor, induce ATP production deficiency, increase calcium overload, and effectively trigger the synergistic apoptosis of lung cancer cells [26]. Also, the use of powerful antioxidants to reduce oxidative stress has been used to prevent cancer [27]. Comprehensive study of the elevated ROS in cancer cells, ROS-regulated signaling pathways, and identification of specific antioxidants as targets could develop the selective and effective therapies for cancer cells [27]. Moreover, direct production of ROS is also one of the mechanisms for common anticancer therapies [28]. Therefore, oxidative stress can cause DNA damage, internal environmental homeostasis damage, and tumorigenicity; and, in turn, oxidative stress pathways might be therapeutic targets for treatment of cancers.

This study analyzed the association of genes in major mitochondrial energy metabolism pathways, extracted from TCGA database of lung cancers, with mutation and clinical characteristics of lung cancers, established the poor prognosis model based on these MMRGs in lung cancers, and further verified the accuracy of this poor prognostic model in lung cancers. These findings will be the promising data for patient stratification, precise prognostic assessment, and personalized treatment of lung cancers.

## 2. Materials and Methods

### 2.1. Dataset and Data Processing

MMRGs were obtained from KEGG PATHWAY database (https://www.kegg.jp/kegg/pathway.html). The related metabolic pathways included glucose oxidative metabolism, fat catabolism, ketone body catabolism, tricarboxylic acid cycle, and oxidative phosphorylation. The gene data of these MMRGs were extracted from The Cancer Genome Atlas (TCGA) database of lung cancers, including transcriptomic, mutation, and complete clinical data of lung cancers. Ensembl of genes was converted with two methods—“clusterProfiler” package transformation, and the online software “ensembl” for the conversion (http://asia.ensembl.org/biomart/martview/e758aa05a6867dc85bfb32852349816b).

### 2.2. GSEA Enrichment Analysis

The gene set enrichment analysis (GSEA) analysis was performed with GSEA software (http://www.gsea-msigdb.org/gsea/index.jsp). GSEA was a computational method that determined whether a priori defined set of genes showed statistically significant and concordant differences between control and experimental groups. First, the transcriptomic data of lung cancer adjacent control tissues were compared to lung cancer tissues, and GSEA analysis was performed to find out the enriched pathways with statistical significance. Second, GSEA analysis was performed with comparison of transcriptomic data between stages I and II of lung cancer tissues. The MMRG SYMBOL was converted to ENTREZID form (https://asia.ensembl.org/index.html) to input GSEA software; then, GSEA analysis was performed in the gseGO (Gene Set Enrichment Analysis of Gene Ontology) model.

### 2.3. Mutation Analysis of MMRGs

The software package “maftools” was used for mutation analysis of MMRGs to identify mutation frequency and characteristics of gene mutations. Lung cancer mutation data in the TCGA database are the mutation annotation format (MAF) data. “maftools” can effectively analyze, annotate, and visualize the mutation data in the MAF format. The long-term survival group means that the survival time was >1000 days. The short-term survival group means that the survival time was <1000 days. The mutation rates of genes in two different survival groups were compared with Student *t*-test between two independent samples.

### 2.4. Correlation Analysis between Age and MMRG Mutation Frequency and between Age and MMRG Expressions

To explore whether MMRGs are associated with age, Pearson correlation analysis was performed between age and MMRG mutation frequency and between age and MMRG expressions, with statistical significance of *p* < 0.05.

### 2.5. Differential Expression Analysis of MMRGs

The flexible and powerful Edger software package was used for differential expression analysis of MMRGs. The popular quasi-likelihood *F*-test was used to determine the significance level. Due to the large amount of RNA-seq data for lung cancer, the quasi-likelihood method was used because it provided a stricter error rate control with consideration of the uncertainty in the dispersion estimation. Each differentially expressed MMRG was determined with the ratio change >2 or <0.5, and *p* value < 0.05.

### 2.6. Functional Enrichment Analysis of Differentially Expressed MMRGs

For Gene Ontology (GO) enrichment analysis, the “clusterProfiler” software package was used to explore the enrichment degree of biological process (BP), cellular component (CC), and molecular function (MF) of the differentially expressed MMRGs. Data visualization was performed with dotplot function.

### 2.7. Survival Analysis of Differentially Expressed MMRGs

The survival time and status of lung cancer patients were extracted from the TCGA database. Univariate and multivariate analyses were carried out for the screened differentially expressed MMRGs. According to the median value of each differentially expressed MMRG, lung cancer patients were divided into high- and low-value groups. Kaplan-Meier survival analysis based on log-rank method was used to analyze each differentially expressed MMRG, with the “survminer” and “survival” R-language packages. Multivariate Cox regression method was used to further determine the factors of poor prognosis.

### 2.8. Immunohistochemical Analysis of Survival-Related Differentially Expressed MMRGs

The immunohistochemical staining results of the proteins corresponding to survival-related differentially expressed MMRGs were obtained from The Human Protein Atlas database (https://www.proteinatlas.org/). The human protein atlas consists of six separate sections (tissue atlas, single cell type atlas, pathology atlas, blood atlas, brain atlas, and cell atlas), each of which focused on a specific aspect of the whole genome analysis of human proteins. The tissue atlas can be used to screen the selected genes for expression in normal lung tissue at the protein level.

### 2.9. Verification of Survival-Related Differentially Expressed MMRGs with Different Datasets

The expressions of survival-related differentially expressed MMRGs between lung cancer and normal groups, as well as between different tumor progression stages, were also tested with different datasets from the online website GEPIA2 (http://gepia2.cancer-pku.cn/#index), this online website included the data in cancer group from the TCGA database, and the data in normal group from the TCGA and GTEX database. Moreover, another datasets including 21 lung cancers and 21 controls (GSE21933 data) from the GEO database were used to verify the expressions of survival-related differentially expressed MMRGs, with the online analysis software GEO2R (https://www.ncbi.nlm.nih.gov/geo/geo2r/?acc=GSE21933).

### 2.10. Protein-Protein Interaction among Differentially Expressed MMRGs

The proteins corresponding to differentially expressed MMRGs were used to construct protein-protein interaction (PPI) network with the STRING database. The Cytohubba plug-in in Cytoscape 3.7.2 software was used to analyze the PPI network to obtain hub molecules [29]. The maximal clique centrality (MCC) algorithm was used to obtain the top 10 genes as the hub genes [29].

### 2.11. Establishment a Model for Diagnosis of Lung Cancer

To establish a mathematical model for the diagnosis of lung cancer, 43 differentially expressed MMRGs were further screened by ridge regression and logistic regression method, and differentially expressed MMRGs with *p* < 0.01 were included in subsequent modeling with Weka 3.85 software. The 10-fold cross-validation was performed to verify the diagnostic performance of this model. The "caret" R-language package was used to calculate the importance of each variable in the logistic regression model.

### 2.12. Western Blotting

Four differentially expressed MMRGs included in logistic regression model were verified with western blot. The fresh lung cancer tissues (*n* = 5) and their corresponding adjacent control tissues (*n* = 5) were collected after surgery in Tai'an central hospital, China. The proteins were extracted and quantified with bicinchoninic acid (BCA) assay method. Five cancer protein samples were equally mixed as tumor protein samples, and five adjacent control protein samples were equally mixed as control protein samples. ALDH18A1 primary antibody was purchased from Beijing Boasen Biotechnology Co., Ltd. (http://www.bioss.com.cn/), and the primary antibodies against ACADL, PPARG, and CPT1B were purchased from Wuhan Abclonal company (https://abclonal.com.cn/). All primary antibodies were rabbit antihuman antibodies. The second antibody was goat antirabbit antibodies. Briefly, an amount (40 *μ*g) of proteins (tumor; control) were separated with one-dimensional 10% PAGE electrophoresis and then were transferred to the PVDF membrane. The proteins on the PVDF membrane were incubated with primary antibody (ALDH18A1, ACADL, PPARG, or CPT1B) and then incubated with secondary antibody, followed by visualization. ACTIN was used as internal reference for western blotting analysis.

### 2.13. Statistical Analysis

For differentially expressed MMRG, GSEA, GO enrichment, KEGG, and western blot analyses, the statistical significance was set as *p* < 0.05.

## 3. Results

### 3.1. MMRG Landscape in Lung Cancer

In total, 188 MMRGs were obtained from the KEGG pathway database, which were analyzed between lung cancer (*n* = 1006) and control lung (*n* = 107) tissues from the TCGA database (Supplementary Table 1).

### 3.2. MMRG-Mediated Signaling Pathways

GSEA enrichment analysis of 188 MMRGs found 151 statistically significant gene sets including 76 gene sets (nominal *p* value < 1%) in lung cancer tissues and 19 statistically significant gene sets including 14 gene sets (nominal *p* value < 1%) in control tissues. Among them, lipid metabolism pathways were mainly enriched in the control lung group (Supplementary Figure 1), and ATP energy generation-related pathways (for example, oxidative phosphorylation, ATP electron transport chain, lactic acidosis, and acid-base balance disorder pathways) were mainly enriched in the lung cancer group (Supplementary Figure 2). These findings clearly demonstrated mitochondrial energy metabolism pathway changes in lung cancer tissues.

Moreover, GSEA enrichment analysis of 188 MMRGs was also performed for lung cancer stages I (*n* = 592 patients) and II (*n* = 385 patients). An interesting result was that lipid metabolism gene set was mainly enriched in stage I (Supplementary Figure 3a), and ATP energy generation process, oxidative phosphorylation pathway, and cell respiration gene sets were mainly enriched in stage II (Supplementary Figures 3b and 3c). These findings also clearly demonstrated mitochondrial energy metabolism pathway changes in advanced stage of lung cancer tissues.

### 3.3. Mutation Status of MMRGs in Lung Cancer

**Figure 1:**
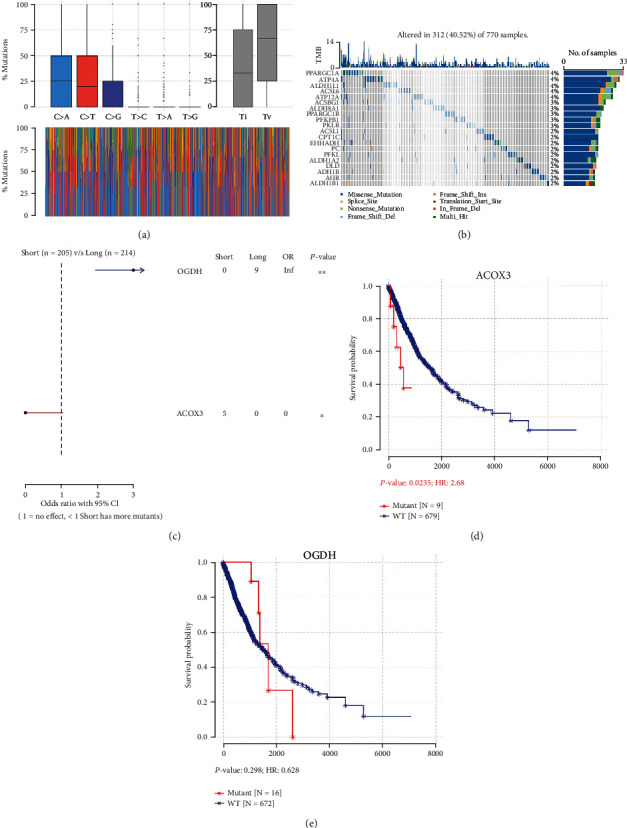
Mutation analysis of 188 mitochondrial energy metabolism pathway-related genes (MMRGs). (a) Mutated genes, change of base distribution. Different colors represent different base mutations. Blue means that base C is mutated to base A. Red means that base C is mutated to base T. Dark blue means that base C is mutated to base G. (b) Oncoplots of 188 MMRGs in lung cancer tissues. (c) Comparison of mutation frequency of different MMRGs in long survival group (>1000 days) vs. short survival group (<1000 days). (d) The survival of the ACOX3 mutant group was better than that of the wild-type group. (e) The survival of the OGDH mutant group was not significant than that of the wild group.

Mutation analysis of 188 MMRGs in lung cancer found that almost all mutated bases were cytosine, and the first three high-mutation bases were C➔A, C➔T, and C➔G (Figure 1(a)). These findings showed that cytosine was very unstable, and the amino group of cytosine was easily oxidized. In addition, the overall mutation rate of MMRGs was not high, and the highest mutation rate was only 4% (Figure 1(b)). Moreover, mutation analysis of 188 MMRGs between the short-survival group (<1000 days) and long-survival group (>1000 days) found that the mutation rate of ACOX3 was significantly higher in the short-survival group relative to the long-survival group, and that the mutationrate of OGDH was significantly higher in long-survival group relative toshort-survival group (*p* < 0.05, Supplementary Table 2, Figure 1(c)). The survival analysis was significant between mutation and wild-type groups of ACOX3 in lung cancers, which was associated with a worse prognosis of lung cancer (*p* < 0.05, Figure 1(d)). The survival analysis was not significant between mutation and wild-type groups of OGDH in lung cancers (*p* > 0.05, Figure 1(e)).

### 3.4. Associations of Age with Expression Level and Mutation Frequency of MMRGs in Lung Cancers

For the lung cancer group, correlation analysis between age and MMRGs found that no any MMRGs were significantly related to age (*p* > 0.05), with a correlation coefficient ranged from -0.1 to 0.1 (Supplementary Table 3). For the control group, correlation analysis between age and MMRGs found that five MMRGs were relatively weak associated with age, with a correlation coefficient greater than 0.2 (Supplementary Table 4). These findings clearly demonstrated that age did not affect MMRGs in lung cancer. Moreover, no statistical significance in mutation frequency was found between younger (<60 years) and older (>60 years) patients, which suggested that age did not affect mutation frequency of MMRGs in lung cancer.

### 3.5. Differentially Expressed MMRGs in Lung Cancer

**Figure 2:**
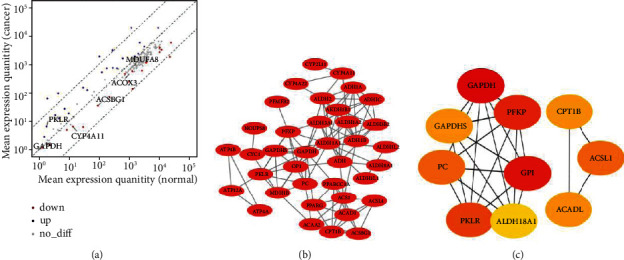
Differentially expressed MMRGs in lung cancers. (a) Differentially expressed MMRGs in lung cancers. The red color indicates low expression and the blue color indicates high expression. (b) Protein-protein interaction (PPI) network constructed with 43 differentially expressed MMRGs. (c) Hub molecules screened by cytohubba.

Among those188 MMRGs, 43 differentially expressed MMRGs were identified between lung cancers and controls, including 24 upregulated and 19 downregulated MMRGs in lung cancers compared to control lung tissues (*p* < 0.05) (Supplementary Table 1; Figure 2(a)). Those 24 upregulated MMRGs were ATP4A, ALDH3B2, ATP4B, ALDH3A1, CYP4A22-AS1, ADH1C, GAPDH, PKLR, PPAT, CPT1B, PFKP, PC, PFKP-DT, ALDH1L1, ADH4, GPI, ALDH8A1, ALDH18A1, ATP12A, GAPDHS, NDUFS6, OXCT2, CYC1, and ALDH1L2; and those 19 downregulated MMRGs were ADH1B, ACADL, ADH1A, CYP4A26P, PPARG, ALDH1A2, ALDH2, ALDH3B1, CYP4A11, MDH1B, ACSBG1, ACSL4, CYP2U1, CYP4A22, CYP4A27P, ACSL1, PPARGC1A, ACAA2, and PFKFB2.

### 3.6. Functional Characteristics of Differentially Expressed MMRGs

GO enrichment analysis of 43 differentially expressed MMRGs found 148 statistically significant BPs, 2 CCs, and 30 MFs (Supplementary Table 5; Supplementary Figure 4). Of them, the significant BPs were mainly involved in small molecule catabolic process, fatty acid metabolic process, and nucleotide metabolic processes (Supplementary Figure 4a). The significant CCs were mainly related to mitochondrial matrix and lipid droplet (Supplementary Figure 4b). The significant MFs were mainly involved in oxidoreductase activity acting on the aldehyde or oxo group of donors, NAD or NADP as acceptor, oxidoreductase activity acting on the aldehyde or oxo group of donors, aldehyde dehydrogenase [NAD(P)+] activity, and aldehyde dehydrogenase (NAD+) activity (Supplementary Figure 4c).

### 3.7. Protein-Protein Interaction Network of Differentially Expressed MMRGs

A total of 43 differentially expressed MMRGs were used to construct the PPI network (Figure 2(b)). Further analysis of this PPI network with Cytoscape found 10 hub molecules, including GAPDH, GAPDHS, PC, PKLR, ALDH18A1, GPI, PFKP, ACADL, ACSL1, and CPT1B (Figure 2(c)). These hub molecules were the crucial enzymes and molecules in glycolysis, TCA, and oxidative phosphorylation. It clearly demonstrated that mitochondrial energy metabolism pathways changed in lung cancer.

### 3.8. Survival Analysis of Differentially Expressed MMRGs in Lung Cancer

**Figure 3:**
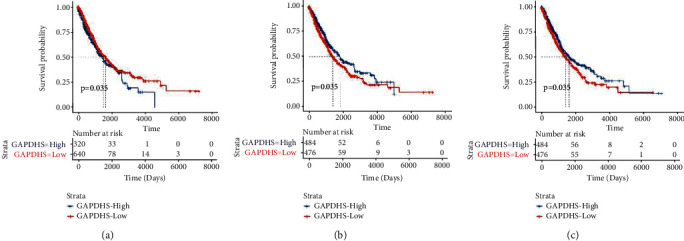
Survival analysis of GAPDHS, ACSBG1, and CYP4A11 in patients with lung cancer. The genetic data of all the tumors were put together and divided into two groups according to the median value.

Survival analysis of 43 differentially expressed MMRGs in lung cancer found that 3 differentially expressed MMRGs (GAPDHS, ACSBG1, and CYP4A11) had survival significance (Supplementary Table 6; Figure 3). (i) The survival prognosis of lung cancer patients was better in the low-expression group than high expression group of GAPDHS (*p* < 0.05). Further, for lung squamous cell carcinomas, the survival prognosis was not statistically significant between low- and high-expression groups of GAPDHS (*p* = 0.094, Supplementary Figure 5a); and for lung adenocarcinomas, the survival prognosis was not statistically significant between low- and high-expression groups of GAPDHS (*p* = 0.39, Supplementary Figure 5a). (ii) The survival prognosis of lung cancer patients was better in the high expression group than low expression group of ACSBG1 (*p* < 0.05). Further, for lung squamous cell carcinomas, the survival prognosis was not statistically significant between low- and high-expression groups of ACSBG1 (*p* = 0.31, Supplementary Figure 5b); and for lung adenocarcinomas, the survival prognosis was better in the high expression group than low expression group of ACSBG1 (*p* = 0.013, Supplementary Figure 5b). (iii) The survival prognosis of lung cancer patients was better in the high expression group than low expression group of CYP4A11 (*p* < 0.05). Further, for lung squamous cell carcinomas, the survival prognosis was significantly better in high expression group than low expression group of CYP4A11 (*p* = 0.02, Supplementary Figure 5c); for lung adenocarcinomas, the survival prognosis was not statistically significant between low- and high expression groups of CYP4A11 (*p* = 0.47; Supplementary Figure 5c).

Moreover, the survival prognosis was significantly better in younger (<60 years) patients than older (>60 years) patients (*p* = 0.025; Supplementary Figure 6a), and was not significant different between lung cancer stages I and II (*p* = 0.77; Supplementary Figure 6b).

Multivariate Cox regression analysis of gender, age, stage, cancer type, GAPDHS, ACSBG1, and CYP4A11 found that older (>60 years) age, highly expressed GAPDHS, low expressed ACSBG1, and low expressed CYP4A11 were high-risk factors for lung cancer prognosis (Supplementary Table 6; Supplementary Figure 7).

### 3.9. Immunohistochemistry Analysis of Survival-Related Differentially Expressed MMRGs in Lung Cancer

**Figure 4:**
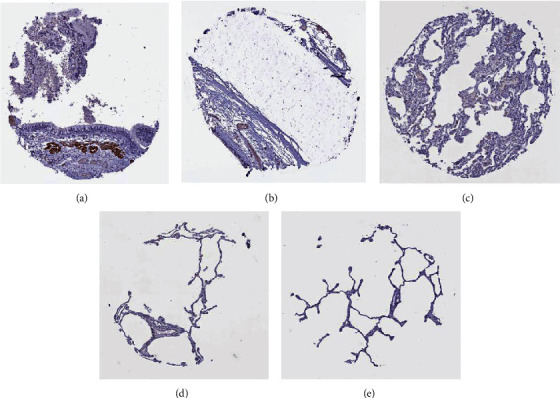
Immunohistochemistry analysis of GAPDHS in lung normal tissues and bronchial tissues.

In the human protein atlas online database, the staining of GAPDHS protein in normal lung tissues was examined. The normal bronchial or lung tissues were stained in 5 subjects, and GAPDHS protein was negative (Figure 4). ACSBG1 protein in 6 normal lung tissues showed moderate staining intensity in alveolar cells or macrophages in lung tissues (Supplementary Figure 8).

### 3.10. Verification of Survival-Related Differentially Expressed MMRGs in Different Datasets of Lung Cancer

**Figure 5:**
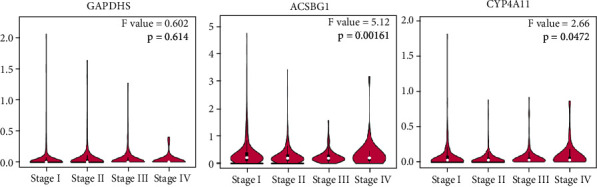
Expressions of survival-related differentially expressed MMRGs (GAPDHS, ACSBG1, and CYP4A11) between different stages of lung cancers.

No significant difference was found between LUAD (*n* = 483) and control (*n* = 347) groups, and between LUSC (*n* = 486) and control (*n* = 338) groups for GAPDHS, ACSBG1, and CYP4A11 (Supplementary Figure 9). There was no significant difference in the expression of GAPDHS in different tumor stages (stages I, II, III, and IV) of lung cancer (*p* > 0.05, Figure 5). However, the expressions of ACSBG1 and CYP4A11 in four tumor stages (stages I, II, III, and IV) were statistically significant difference, respectively (*p* < 0.05, Figure 5). Further verification in the independent data set GSE21933 showed that ACSBG1 was a significantly low expression in the lung cancer group, and LogFC was 0.81 (*p* < 0.05, Supplementary Table 7). There was no significant difference in GAPDHS and CYP4A11 between the lung cancer group and the normal group (*p* > 0.05, Supplementary Table 7).

### 3.11. Construction Machine Learning Model Based on Differentially Expressed MMRGs for Lung Cancer

**Figure 6:**
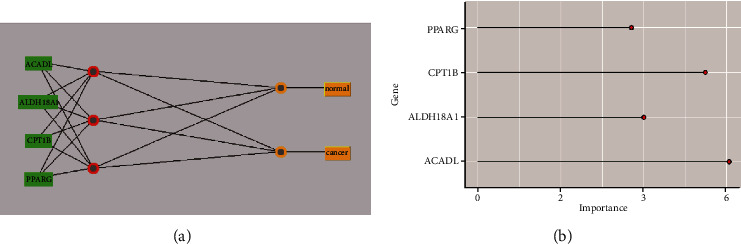
Construction of four-differentially expressed MMRG-signature model in lung cancer. (a) A network model constructed by four differentially expressed MMRGs predicts lung cancer. (b) The importance of four MMRGs in logistic regression models for diagnosis of lung cancer.

Ridge regression was used to further reduce the dimension, and six genes (ACADL, ADH1B, ALDH18A1, CPT1B, CYP2U1, and PPARG) were screened out. Then, four characteristic genes were screened out through logistic regression modeling (Supplementary Table 8). Weka3.8.5 software modeling was used to classify adjacent normal tissues and lung cancer tissues. Four models were established, which were decision stump, logistic regression, naive Bayes, and multilayer perceptron in order (Figure 6(a)). A 10-fold cross-validation shows that the correct classification rate was 96.13% for decision stump model, 98.74% for logistic regression model, 97.57% for naive Bayes model, and 98.29% for multilayer perceptron model. ROC analysis showed that the AUC values of four 4 corresponding models were 0.902, 0.992, 0.990, and 0.992, respectively. The logistic regression model had a missed diagnosis rate of only 0.6%. Also, logical regression formula was *y* = 0.0069∗ACADL − 0.001∗ALDH18A1 − 0.0405∗CPT1B + 0.0008∗PPARG − 1.625. It could be seen the importance of the various variables in the logistic regression model (Figure 6(b)). It was easy to find that the importance of 4 differentially expressed MMRGs in the logistic regression model was ACADL > CPT1B > ALDH18A1 > PPARG.

### 3.12. Verification of Four Differentially Expressed MMRGs in Machine Learning Model with Western Blot

**Figure 7:**
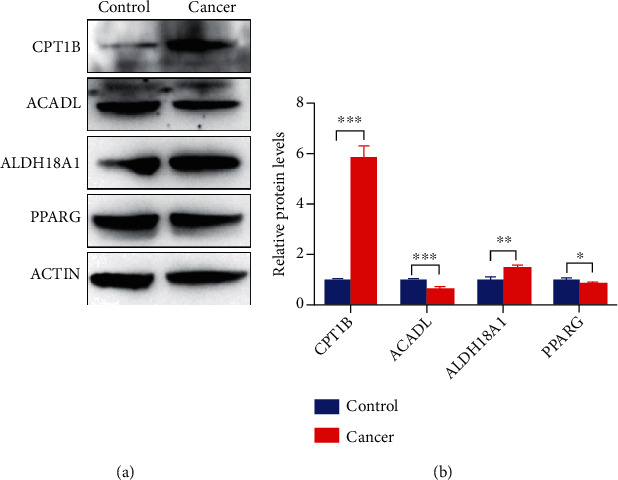
Western blotting analysis of four differentially expressed MMRGs in the signature model. The expression levels of CTP1B and ALDH18A1 protein were significantly upregulated in the tumor group, and the expression levels of ACADL and PPARG protein were slightly downregulated in the tumor group. Control: *n* = 3; and cancer: *n* = 3. ^∗^*p* < 0.05, ^∗∗^*p* < 0.01, and ^∗∗∗^*p* < 0.01.

Western blot showed that, compared to the control group, the expression levels of CPT1B and ALDH18A1 proteins were significantly upregulated (*p* < 0.05), and the expression levels of ACADL and PPARG protein were slightly downregulated with *p* < 0.05 (Figure 7).

## 4. Discussion

Mitochondrial energy metabolism abnormality is the important hallmark of lung cancer. The mitochondrial energy metabolism-related pathways mainly included glycolysis, tricarboxylic acid cycle, oxidative phosphorylation, ketone body metabolism, fat metabolism, and gluconeogenesis. Although glycolysis occurs in cytoplasm, however, glycolysis is the first stage of the aerobic oxidation of sugar, and it was included in this study. Thus, a total of 188 MMRGs in these pathways was used to analyze the alteration landscape and their clinical significance in lung cancers. This study also identified a risk factor for poor prognosis of lung cancer from mitochondrial energy metabolism pathway.

Compared to control lung tissues, 43 differentially expressed MMRGs were identified in lung cancer tissues, including 24 upregulated (ATP4A, ALDH3B2, ATP4B, ALDH3A1, CYP4A22-AS1, ADH1C, GAPDH, PKLR, PPAT, CPT1B, PFKP, PC, PFKP-DT, ALDH1L1, ADH4, GPI, ALDH8A1, ALDH18A1, ATP12A, GAPDHS, NDUFS6, OXCT2, CYC1, and ALDH1L2) and 19 downregulated MMRGs (ADH1B, ACADL, ADH1A, CYP4A26P, PPARG, ALDH1A2, ALDH2, ALDH3B1, CYP4A11, MDH1B, ACSBG1, ACSL4, CYP2U1, CYP4A22, CYP4A27P, ACSL1, PPARGC1A, ACAA2, and PFKFB2).

Survival analysis of 43 differentially repressed MMRGs found 3 survival-related MMRGs (GAPDHS, ACSBG1, and CYP4A11). GAPDHS is an evolutionarily conserved essential enzyme in the glycolytic pathway [30], whose high expression in melanoma is a biomarker of poor prognosis [12]. ACSBG1 is an acyl-CoA synthetase, plays an important role in fatty acid metabolism [13], and is significantly downregulated in psoriatic lesions [14]. CYP4A11 is one member of cytochrome P4504 (CYP4) family involved in the metabolisms of fatty acids, which is downregulated in liver cancer tissues [16] and in clear-cell renal cell carcinoma tumor tissues [31]. Our study found the significantly high expression of GAPDHS, low expression of ACSBG1, and low expression of CYP4A11 in lung cancer tissues predicted poor prognosis for survival, whatever with single-factor and multifactor survival analytical strategies. It clearly emphasized the scientific merits of these three differentially expressed MMRGs in the progression of lung cancer. The prognosis of the ACOX3 mutant group was worse than that of the wild-type group, which suggests that the mutation in ACOX3 is a biomarker of poor prognosis. GSEA enrichment analysis showed that oxidative phosphorylation and ATP energy generation-related pathways were significantly enriched in lung cancer tissues, which may be adapted to the continuous proliferation and invasion of lung cancer cells.

Ridge regression analysis of 43 differentially expressed MMRGs identified six differentially expressed MMRGs (CPT1B, ACADL, PPARG, ALDH18A1, ADH1B, and CYP2U1). These six differentially expressed MMRGs were further analyzed with logistic regression modeling to obtain significantly differentially expressed MMRGs in the logistic regression model. The accuracy of 10-fold cross-validation of logistic regression model in the diagnosis of lung cancer was 98.74%. The missing rate of logistic regression model was only 0.6%. It clearly showed that the performance of logistic regression model was excellent. The study optimized and screened for characteristic differentially expressed MMRGs to establish logistic regression models that are no less accurate in diagnosis of lung cancer than other models [32–34]. It is possible that this model will be developed to screen for clinical lung cancer in the future. CPT1B and ACADL were the most important in the diagnosis of lung cancer in logistic regression models. CPT1B, a rate-limiting step in the catalytic oxidation of fatty acids, is upregulated in prostate cancer and is associated with poor prognosis [35]. ACADL is a mitochondrial enzyme that is frequently downregulated in hepatocellular carcinoma, and its low expression is significantly associated with poor clinical prognosis in hepatocellular carcinoma patients [36]. PPARG is a nuclear receptor regulating adipocyte differentiation [37]. PPARG is one of three members of the PPAR family of transcription factors that influence the function of serine/threonine kinase 3, which can lead to a more aggressive disease phenotype in prostate cancer [38]. By targeting ALDH18A1, GCN2-mediated phosphorylation of eIF2*α* is increased by decreasing intracellular proline levels, thereby inhibiting the development of melanoma [39]. ALDH18A1 has a profound influence on the proliferation, self-renewal, and tumorigenicity of NB cells and is a potential risk factor for NB patients [40]. Some studies have shown that the decrease of ADH1B is associated with human lung cancer [41]. ADH1B was a low expression gene in our study. There are a large number of reports that mathematical models can be used to predict the survival prognosis of cancer patients [42–46], and many factors affect the prognosis. Gene expression can predict good or bad survival, but the exact survival interval is not accurate enough for mathematical models to predict. There are difficulties and challenges in accurately predicting patient survival. However, considerable progress has been made with different methods to diagnose lung cancer. The diagnostic accuracy of lung cancer was 97.3% when some scholars studied the lung cancer tissue sections under weak supervision [47, 48]. It clearly demonstrates that this four-differentially expressed MMRG-signature model has great potential for lung cancer. Furthermore, the results of immunoblotting experiment of these four MMRGs were consistent with the expression trend of their corresponding mRNAs in the logistic regression model, which further confirmed that this model has certain diagnostic performance.

## 5. Conclusion

Mitochondrial energy metabolism pathway alterations were the important hallmarks of lung cancer. This study analyzed the expression levels and mutation status of 188 MMRGs in lung cancer and control tissues, which identified 43 differentially expressed MMRGs (24 upregulated and 19 downregulated) in lung cancers. Three survival-related differentially expressed MMRGs were found, including GAPDHS, ACSBG1, and CYP4A11, in lung cancer. In lung cancer tissue, high expression of GAPDHS, low expressions of ACSBG1, CYP4A11, mutated ACOX3, and old age predict a poor prognosis. Ridge regression analysis of these 43 differentially expressed MMRGs constructed a four-MMRG-signature model (*y* = 0.0069∗ACADL − 0.001∗ALDH18A1 − 0.0405∗CPT1B + 0.0008∗PPARG − 1.625), which has an excellent capability to discriminate lung cancers from controls with the correct classification rate up to 98.74%, AUC value up to 0.992, and a missed diagnosis rate of only 0.6%. It has important scientific merits for diagnosis and prognostic assessment of lung cancer. The expression levels of CPT1B and ALDH18A1 proteins were significantly upregulated, while the expression levels of ACADL and PPARG proteins were slightly downregulated. It showed that these four proteins had certain diagnostic performance for lung cancer.

## Data Availability

All data are present as figures and supplementary materials, which can be available in the journal website.

## Supplementary Materials

Supplementary 1 Supplementary Figure 1: GSEA enrichment analysis of adjacent normal tissues compared with lung cancer group.

Supplementary 2 Supplementary Figure 2: GSEA enrichment analysis of lung cancer tissues compared with the adjacent normal group.

Supplementary 3 Supplementary Figure 3: GSEA enrichment analysis for lung cancer stages I vs. II.

Supplementary 4 Supplementary Figure 4: analysis of the enriched pathways of differentially expressed MMRGs in BP, CC, and MF.

Supplementary 5 Supplementary Figure 5: the survival analysis of 3 differentially expressed MMRGs (GAPDHS, ACSBG1, and CYP4A11) in lung adenocarcinomas and lung squamous cell carcinomas.

Supplementary 6 Supplementary Figure 6: the survival analysis of different subgroups of lung cancer.

Supplementary 7 Supplementary Figure 7: multivariate cox regression analysis of lung cancer.

Supplementary 8 Supplementary Figure 8: immunohistochemistry of normal lung tissues showed staining of ACSBG1.

Supplementary 9 Supplementary Figure 9: the expression of survival-related differentially expressed MMRGs in TCGA and GTEX database of lung cancer.

Supplementary 10 Supplementary Table 1: differential expression analysis of mitochondrial energy metabolism pathway-related genes between lung cancer and control groups.

Supplementary 11 Supplementary Table 2: mutation status of 188 mitochondrial energy metabolism pathway-related genes (MMRGs) in lung cancer and control tissues.

Supplementary 12 Supplementary Table 3: correlation analysis between age and MMRGs in the lung cancer group.

Supplementary 13 Supplementary Table 4: correlation analysis between age and MMRGs in the lung control group.

Supplementary 14 Supplementary Table 5: functional characteristics analysis of 43 differentially expressed MMRGs using GO enrichments.

Supplementary 15 Supplementary Table 6: clinical characteristics of lung cancer patients.

Supplementary 16 Supplementary Table 7: difference analysis of survival-related differentially expressed MMRGs in dataset GSE21933.

Supplementary 17 Supplementary Table 8: four-differentially expressed MMRG-signature model for lung cancer diagnosis.
