# Supplementary figures and images for: Comprehensive Analysis of Alteration Landscape and Its Clinical Significance of Mitochondrial Energy Metabolism Pathway-Related Genes in Lung Cancers
Source: Oxid Med Cell Longev. 2021 Dec 20;2021:9259297. doi: 10.1155/2021/9259297 (PMC8713050; doi:10.1155/2021/9259297)

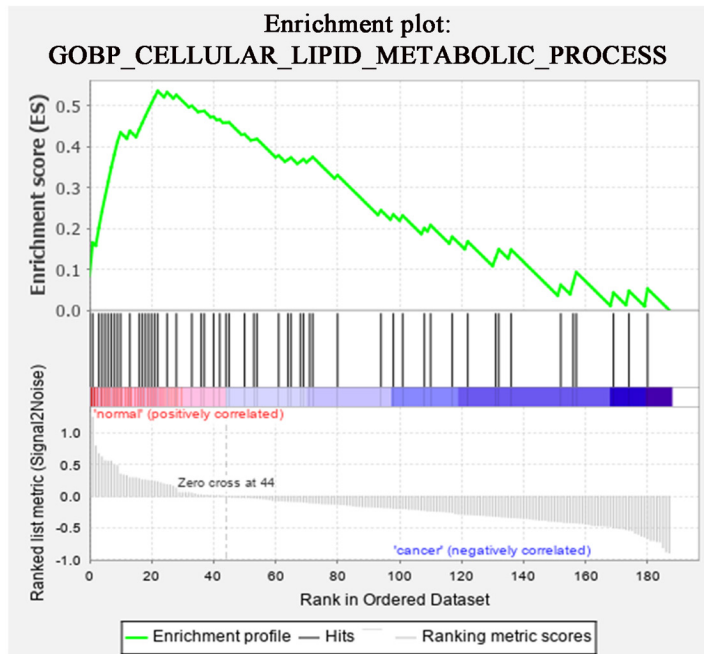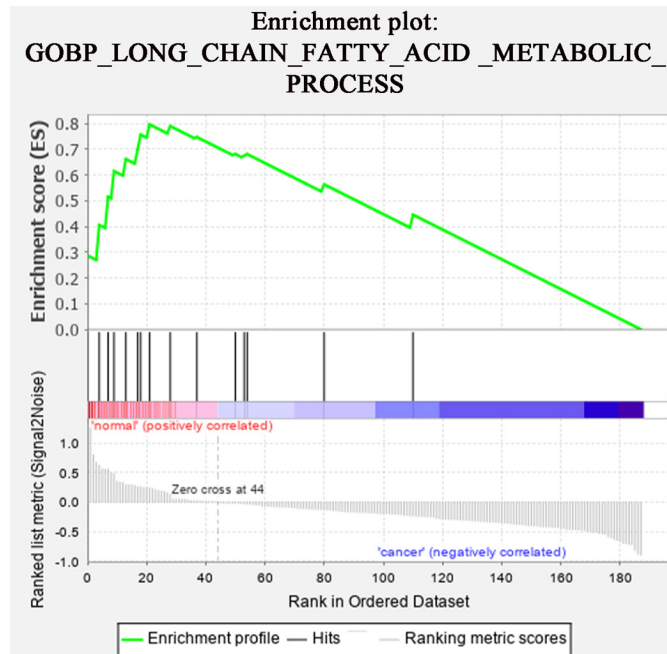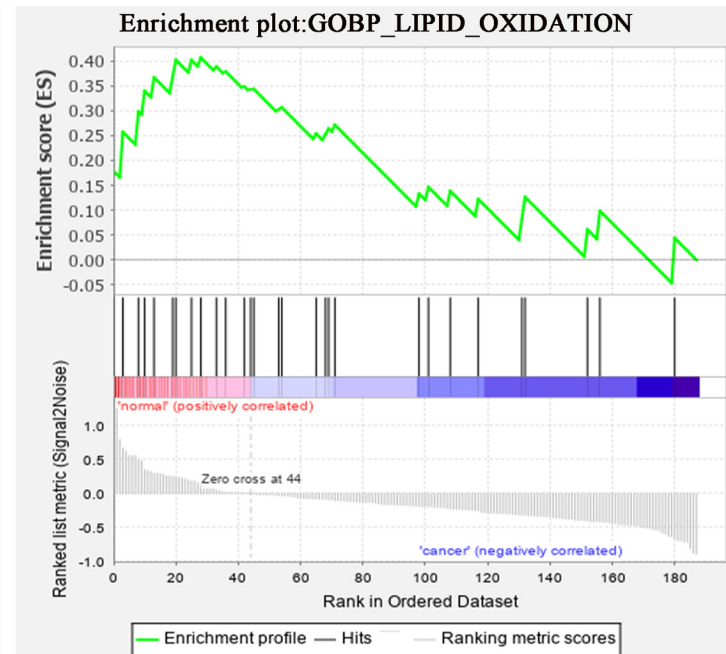

GSEA enrichment analysis of adjacent normal tissues compared with lung cancer group.

Supplement: Supplementary 1 — Supplementary Figure 1: GSEA enrichment analysis of adjacent normal tissues compared with lung cancer group. [file 9259297.f1.pdf]

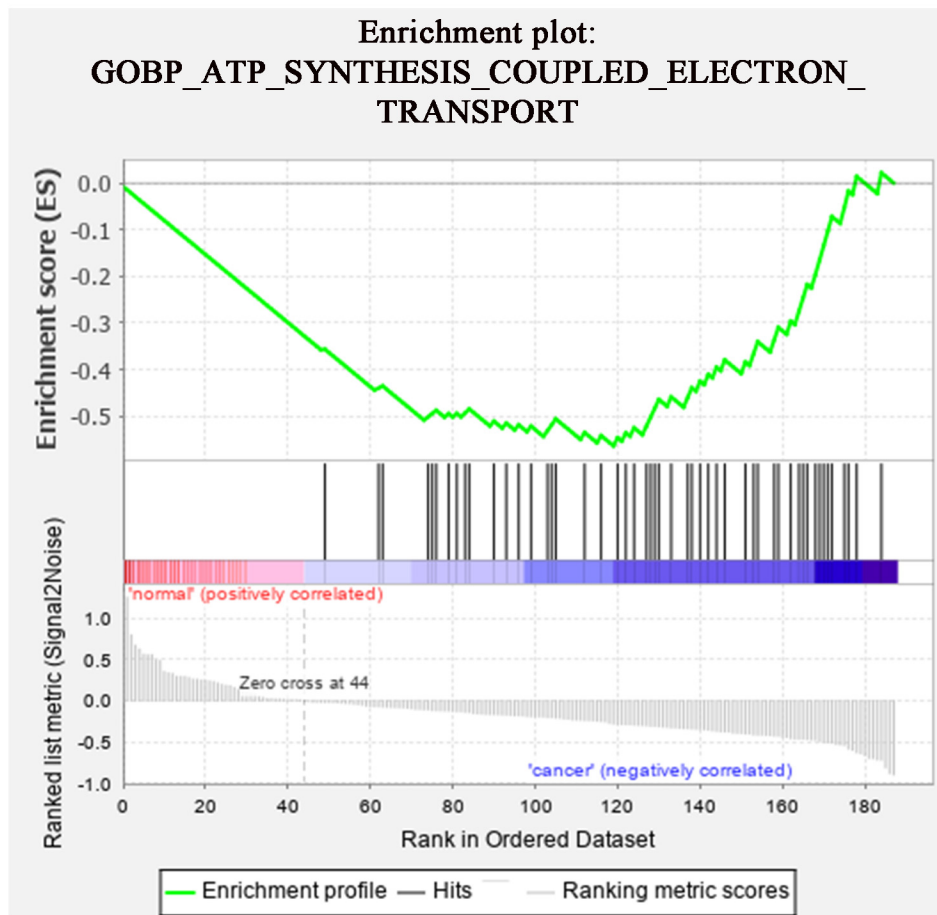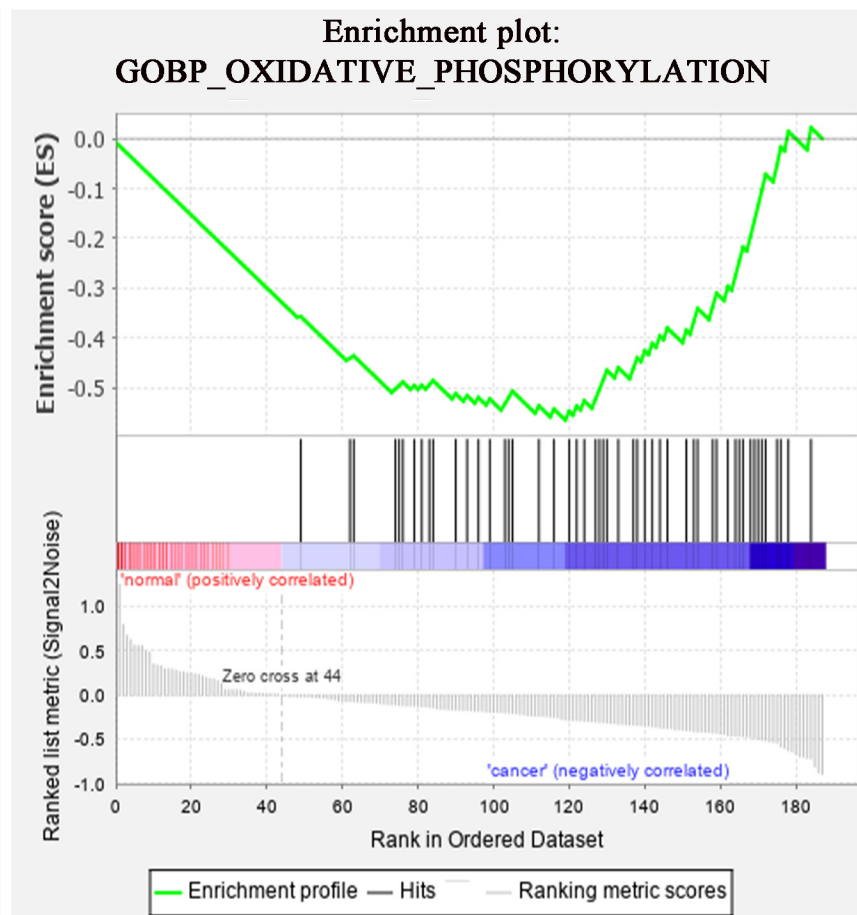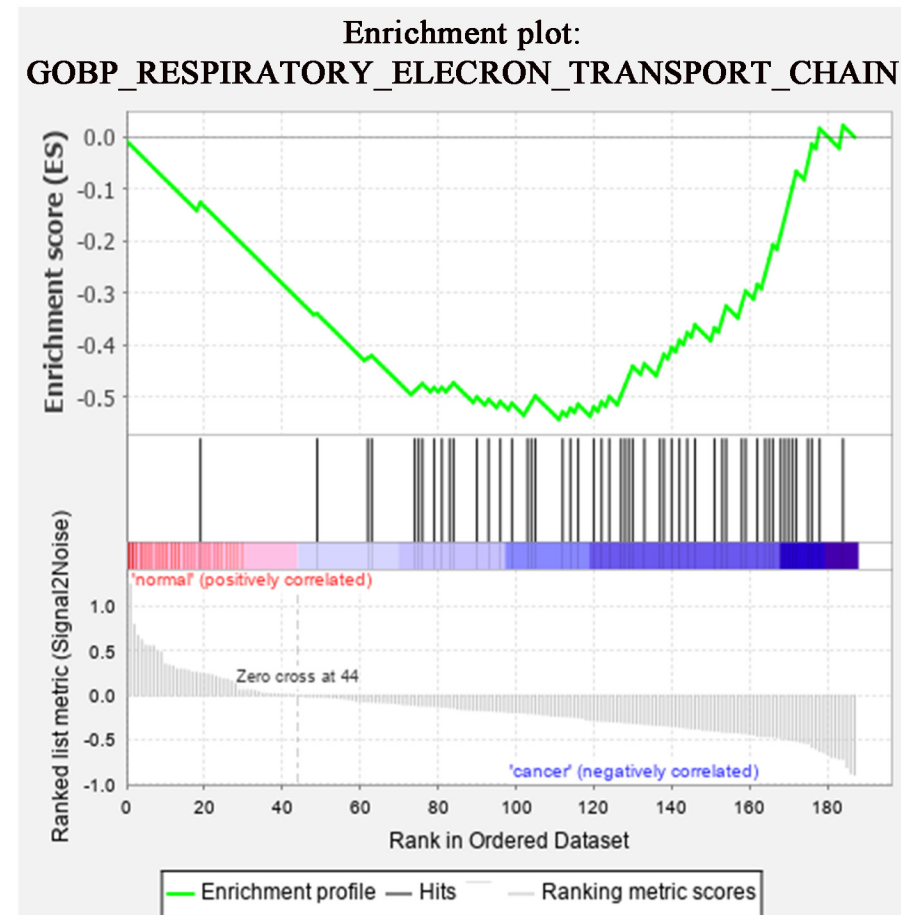

GSEA enrichment analysis of lung cancer tissue compared with the adjacent normal group.

Supplement: Supplementary 2 — Supplementary Figure 2: GSEA enrichment analysis of lung cancer tissues compared with the adjacent normal group. [file 9259297.f2.pdf]

Analysis of the enrichment pathway of differentially expressed MMRGs in BP,CC and MF.

a

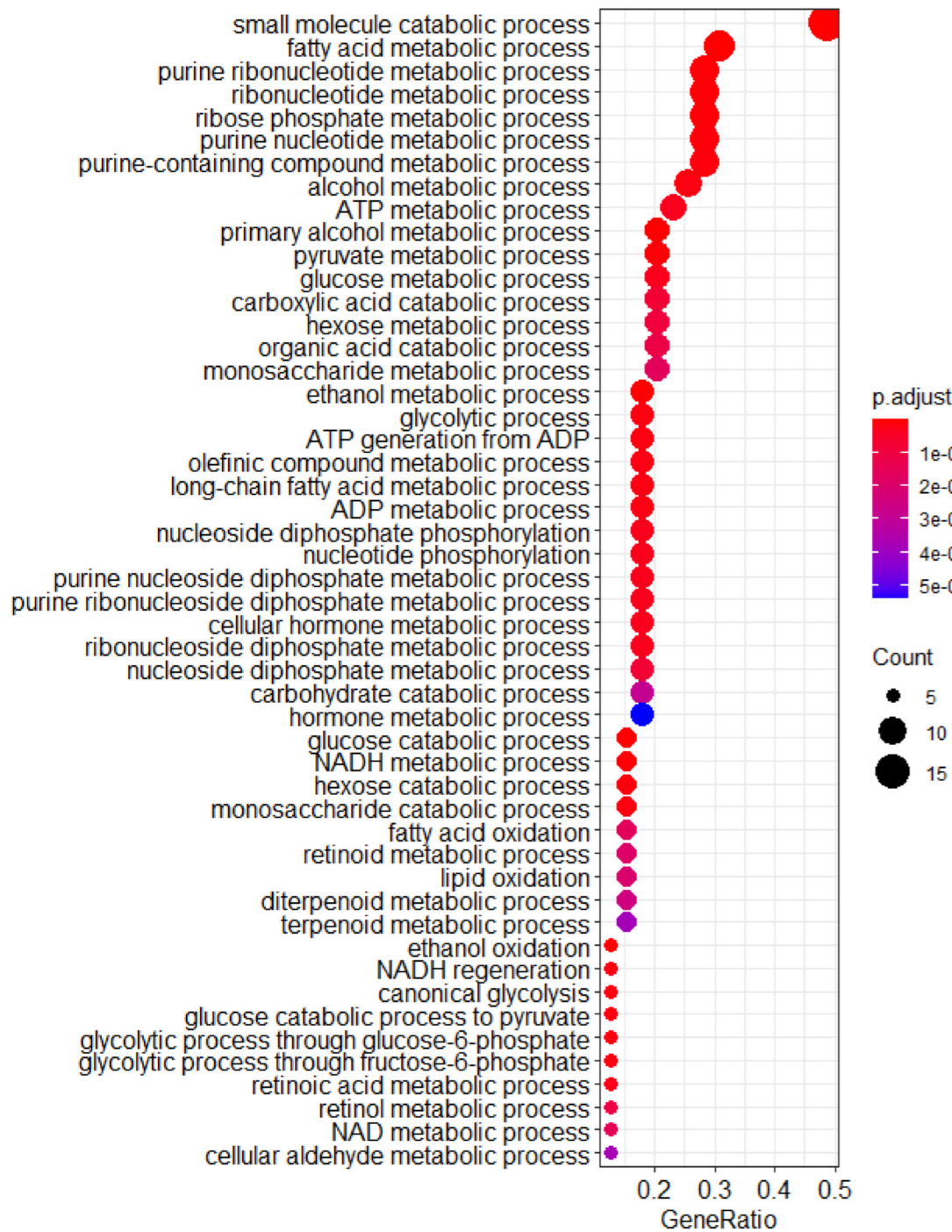

b

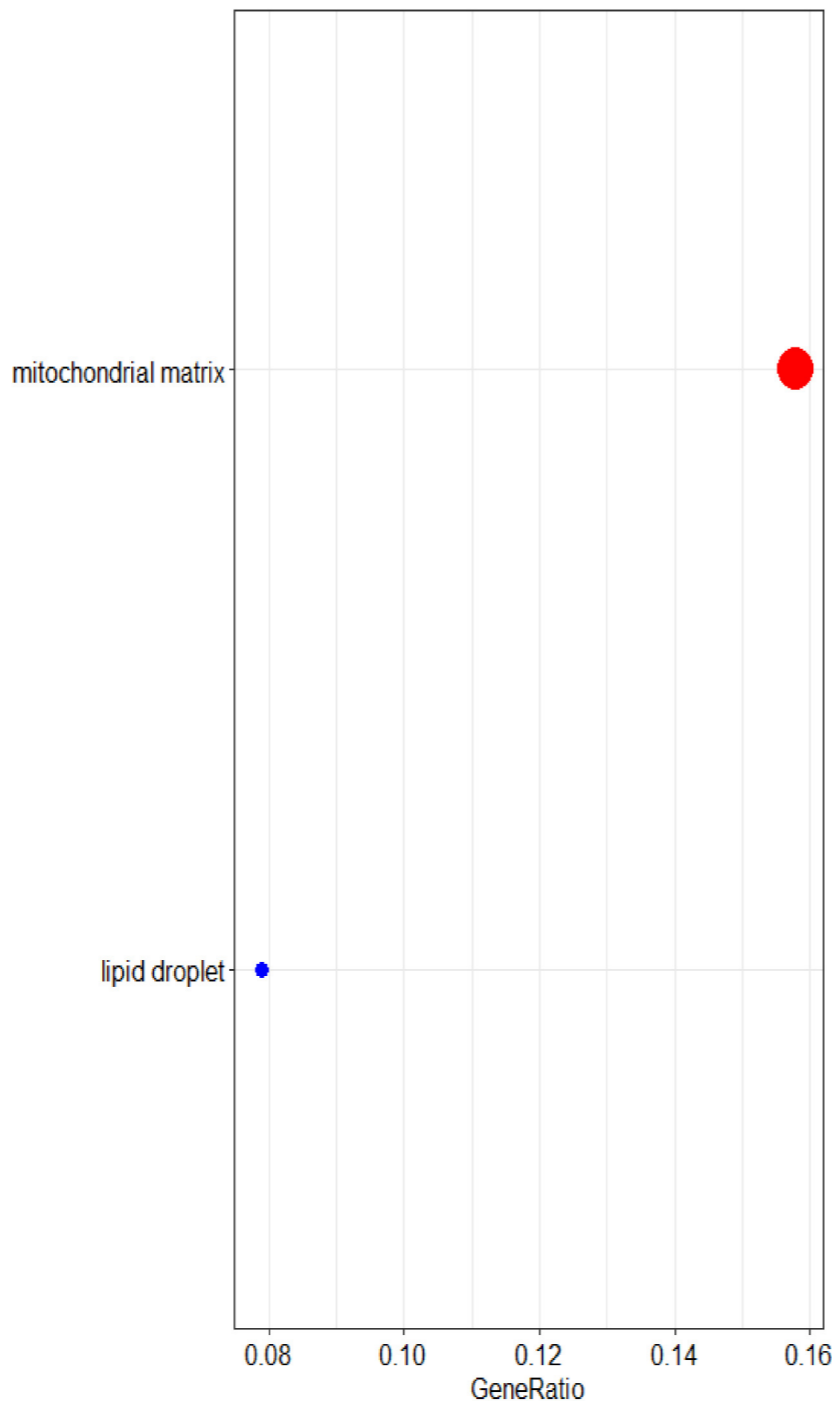

c

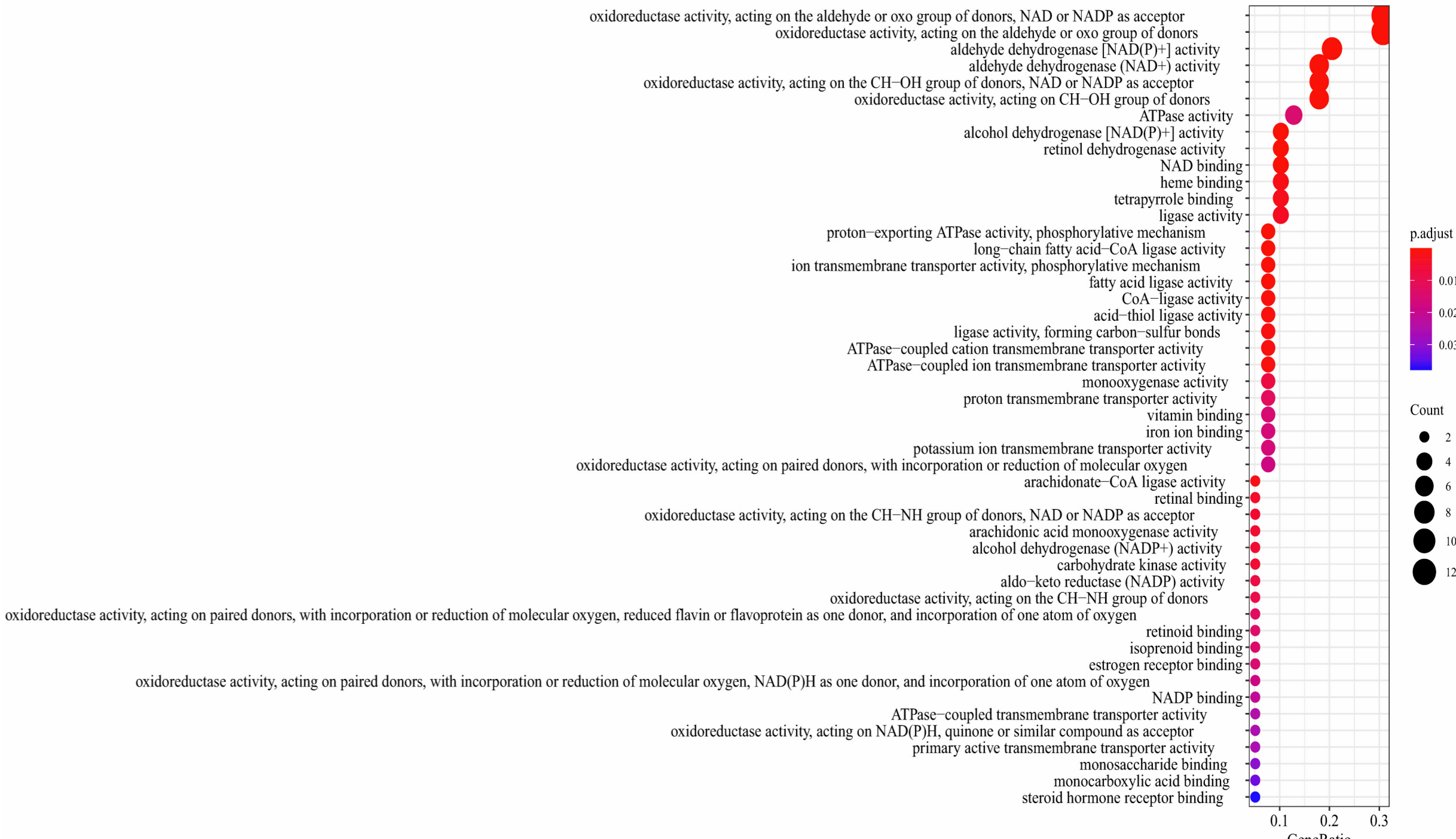

Supplement: Supplementary 4 — Supplementary Figure 4: analysis of the enriched pathways of differentially expressed MMRGs in BP, CC, and MF. [file 9259297.f4.pdf]

a.

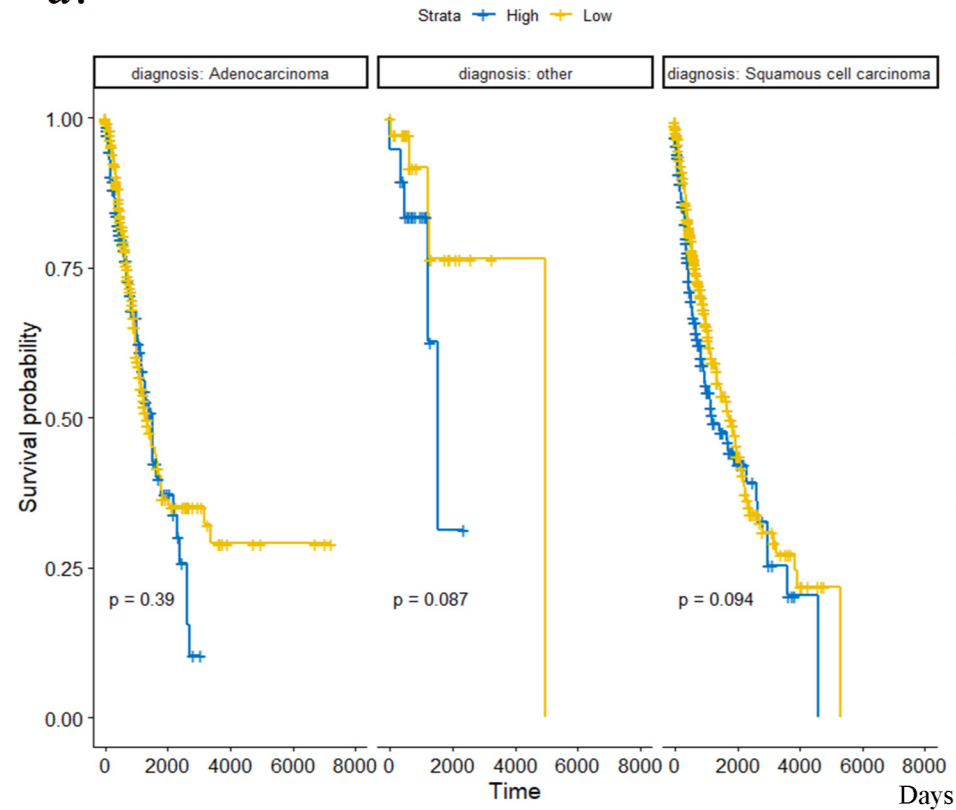

b.

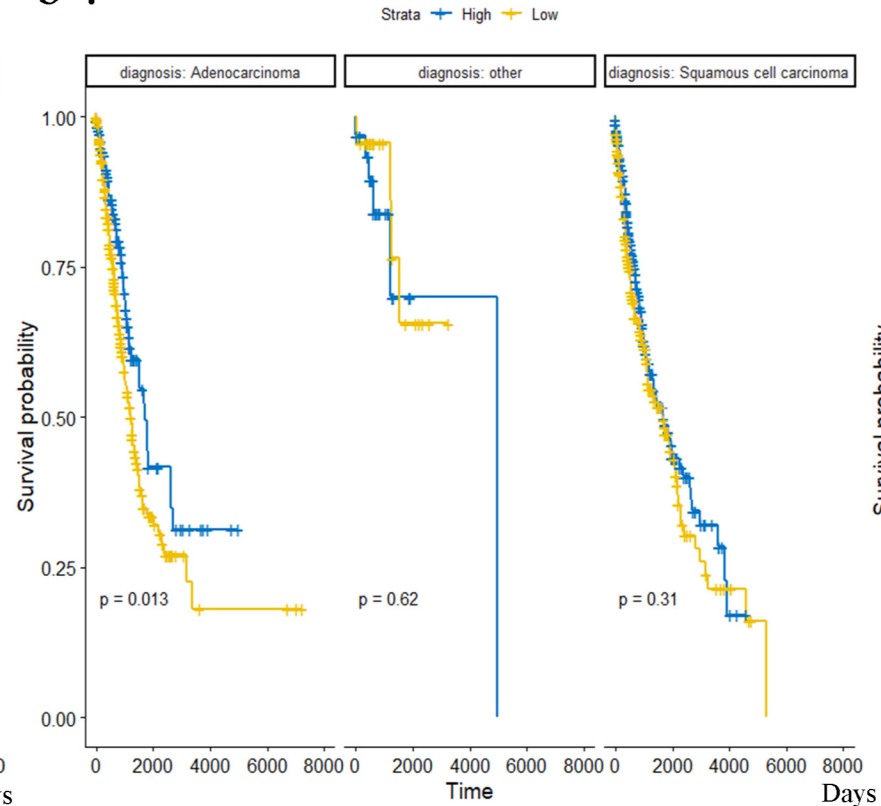

c.

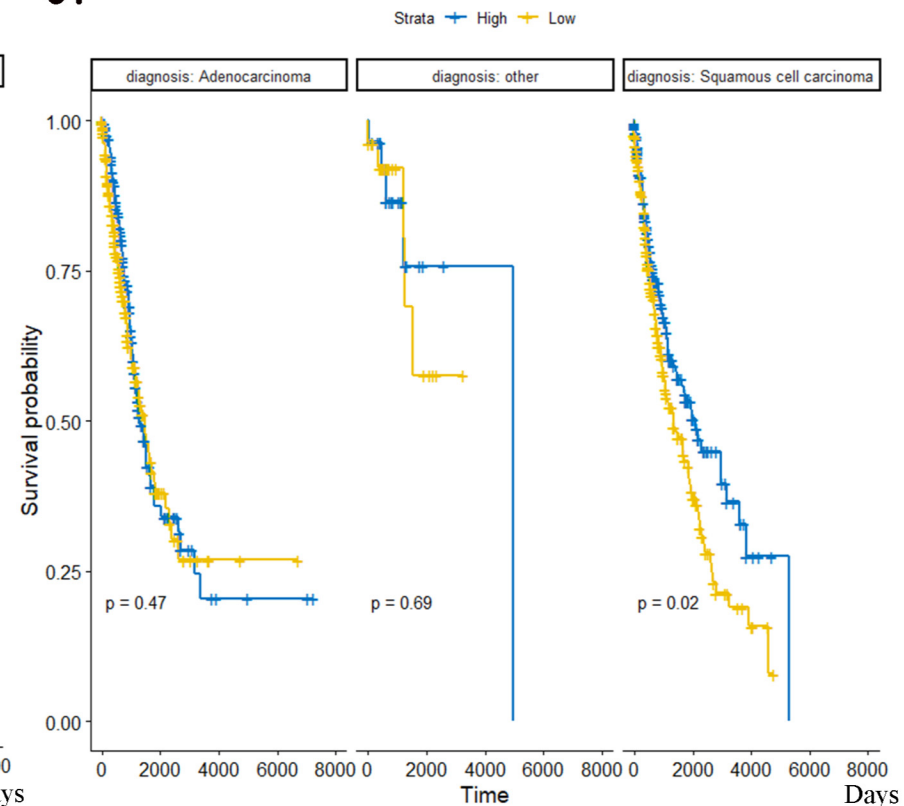

Survival analysis of three genes in lung cancer subgroup

Supplement: Supplementary 5 — Supplementary Figure 5: the survival analysis of 3 differentially expressed MMRGs (GAPDHS, ACSBG1, and CYP4A11) in lung adenocarcinomas and lung squamous cell carcinomas. [file 9259297.f5.pdf]

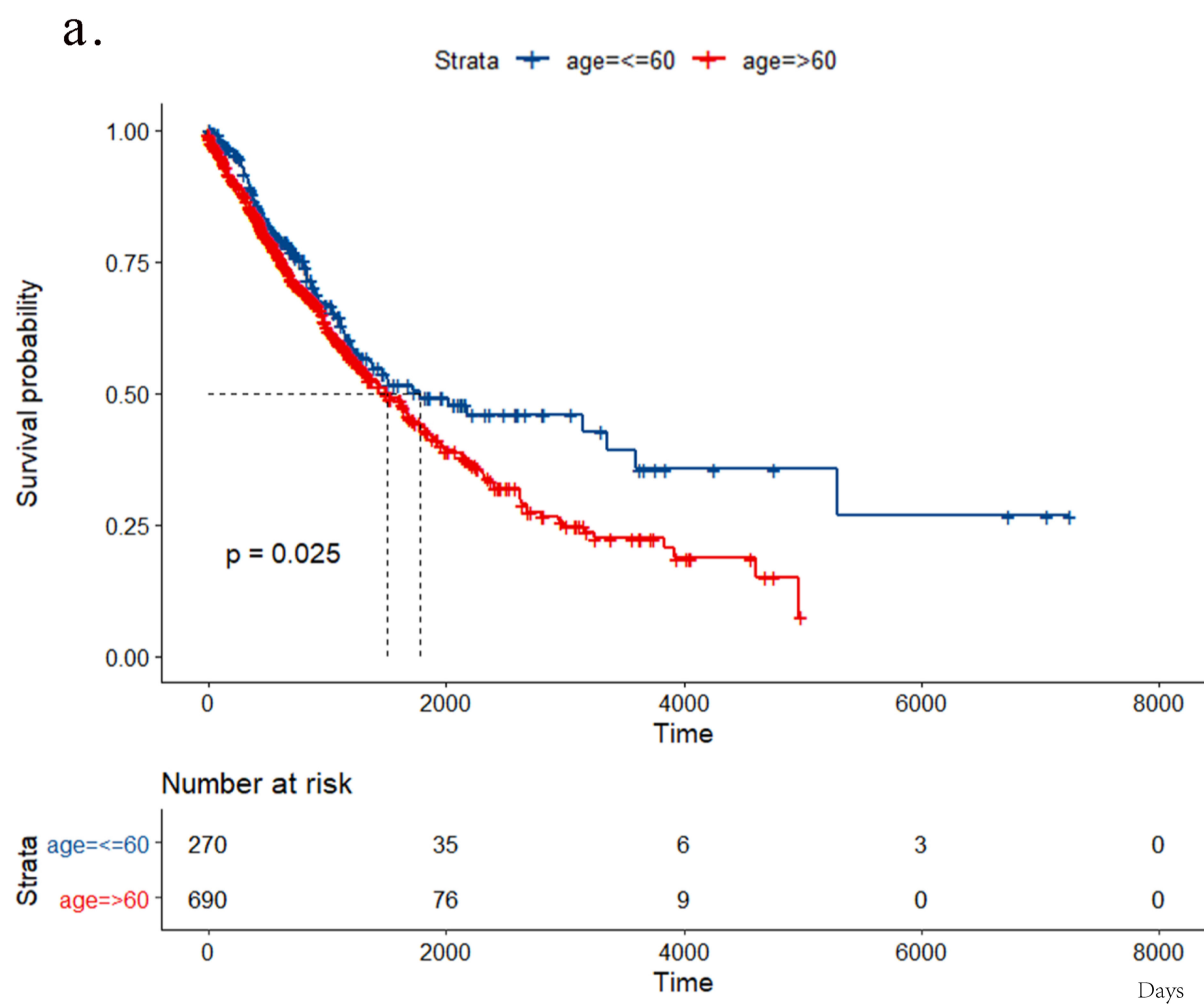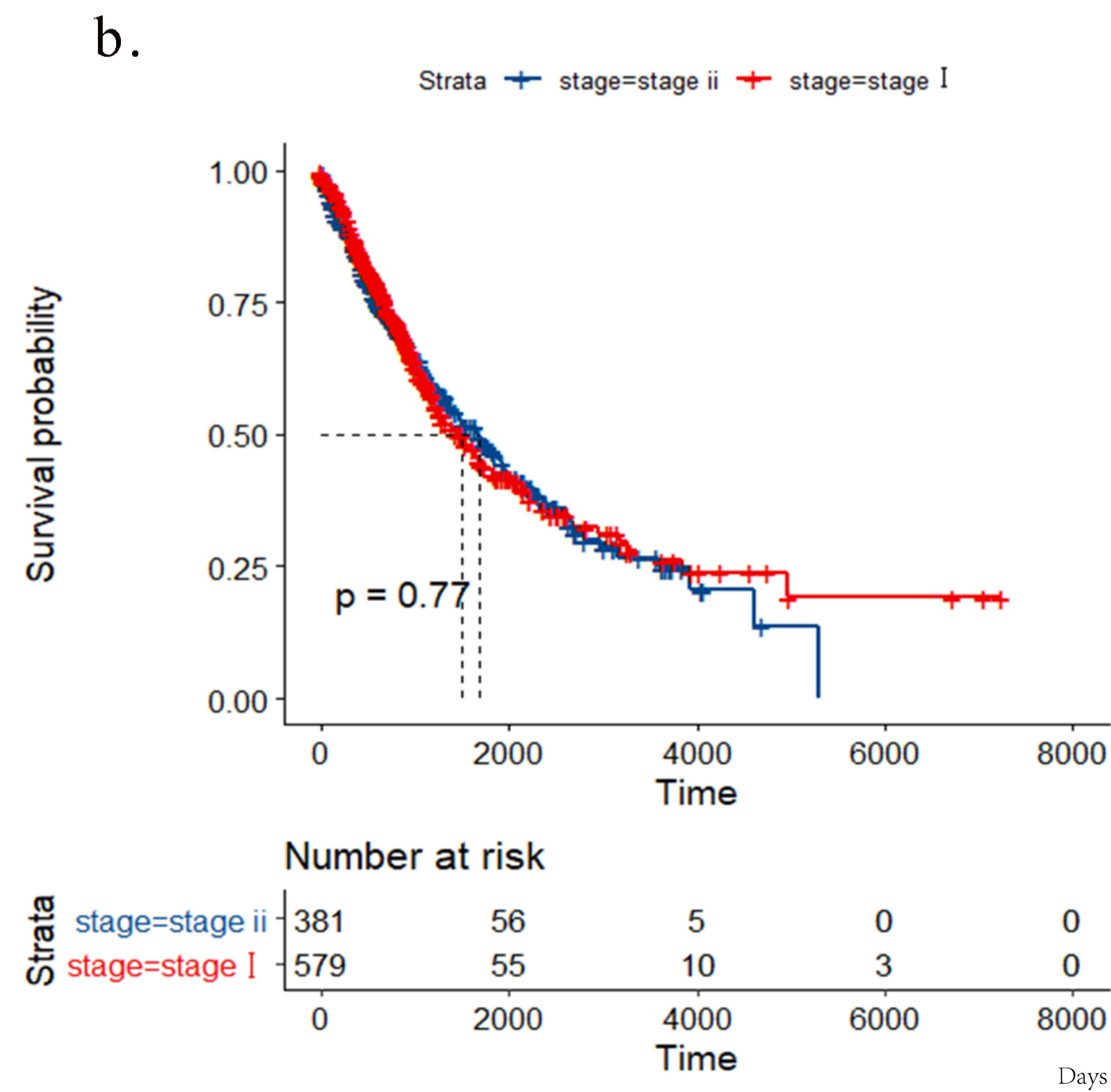

Survival analysis of different subgroups of lung cancer .

Supplement: Supplementary 6 — Supplementary Figure 6: the survival analysis of different subgroups of lung cancer. [file 9259297.f6.pdf]

# Hazard ratio

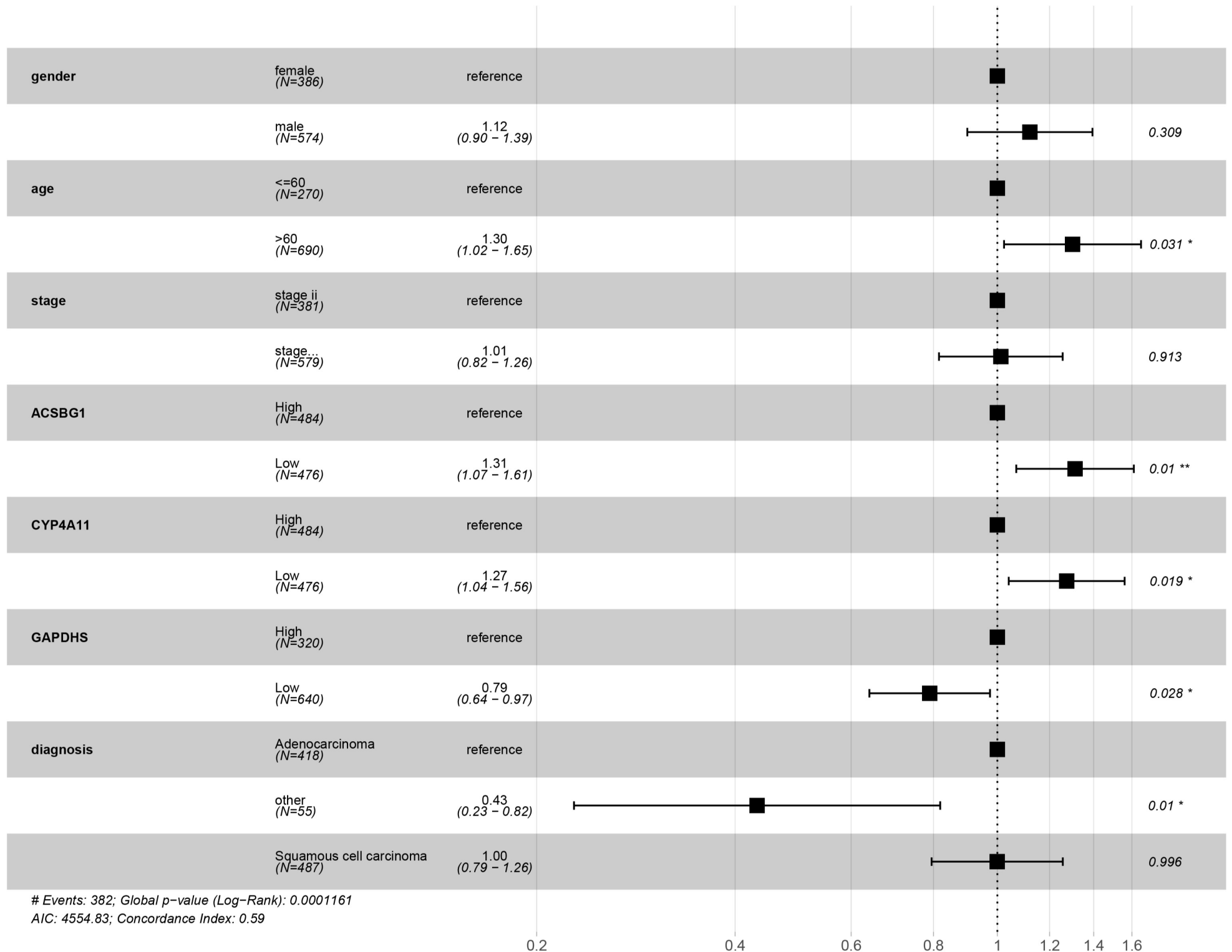

Cox multivariate survival analysis of lung cancer.

Supplement: Supplementary 7 — Supplementary Figure 7: multivariate cox regression analysis of lung cancer. [file 9259297.f7.pdf]

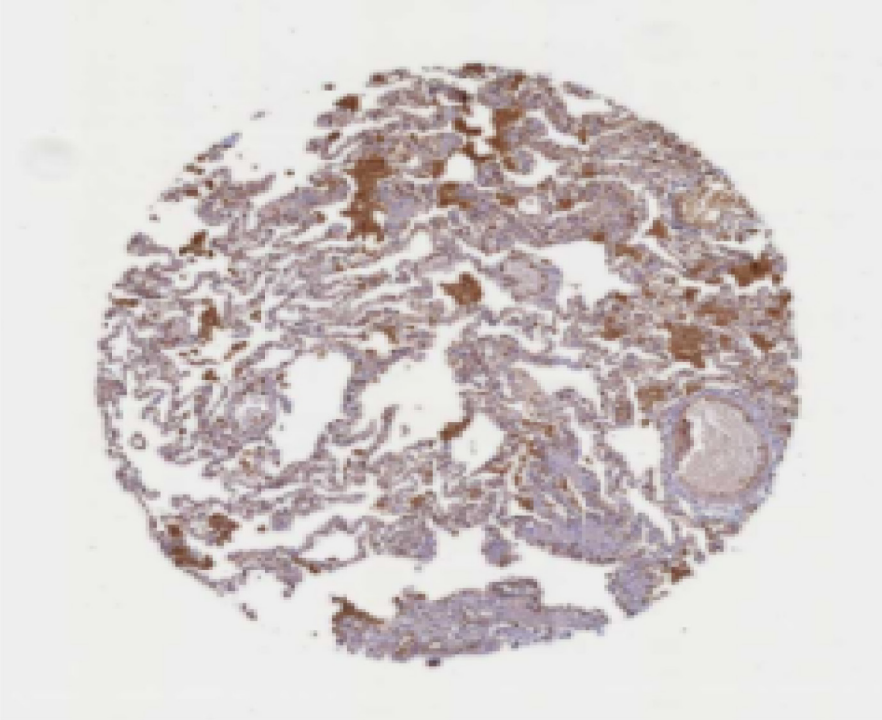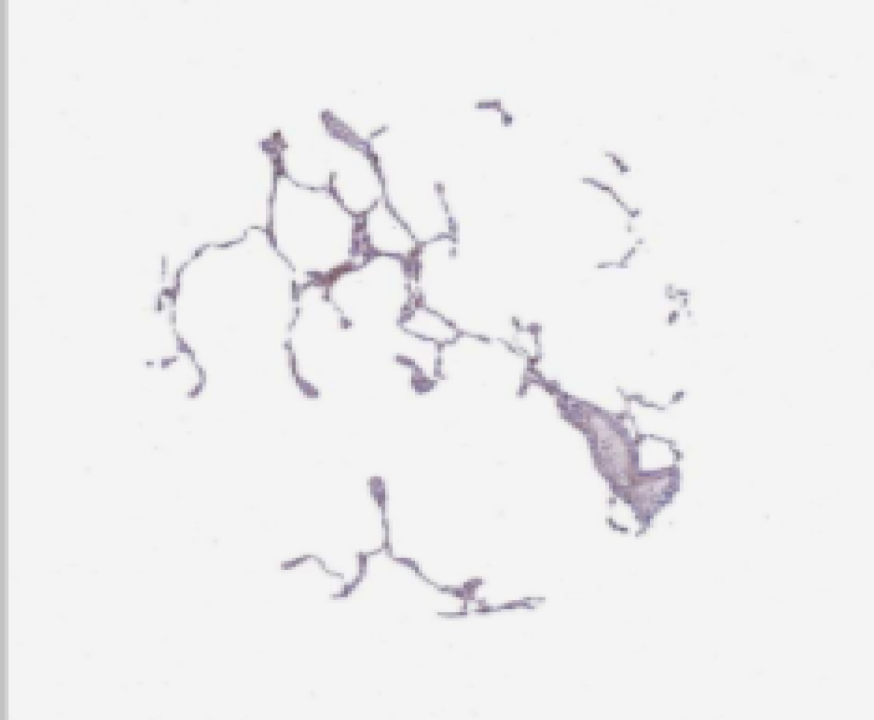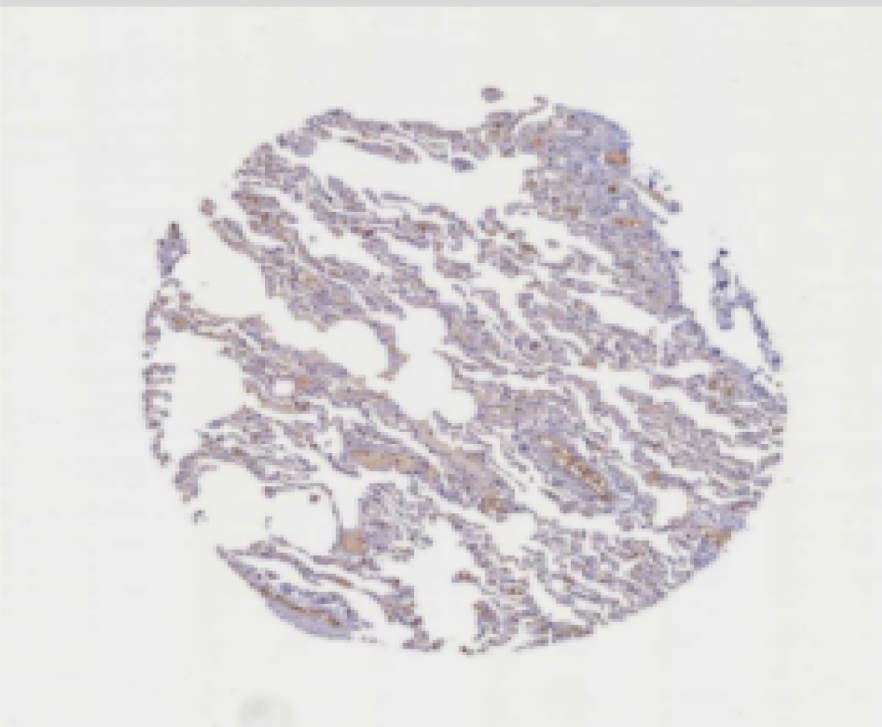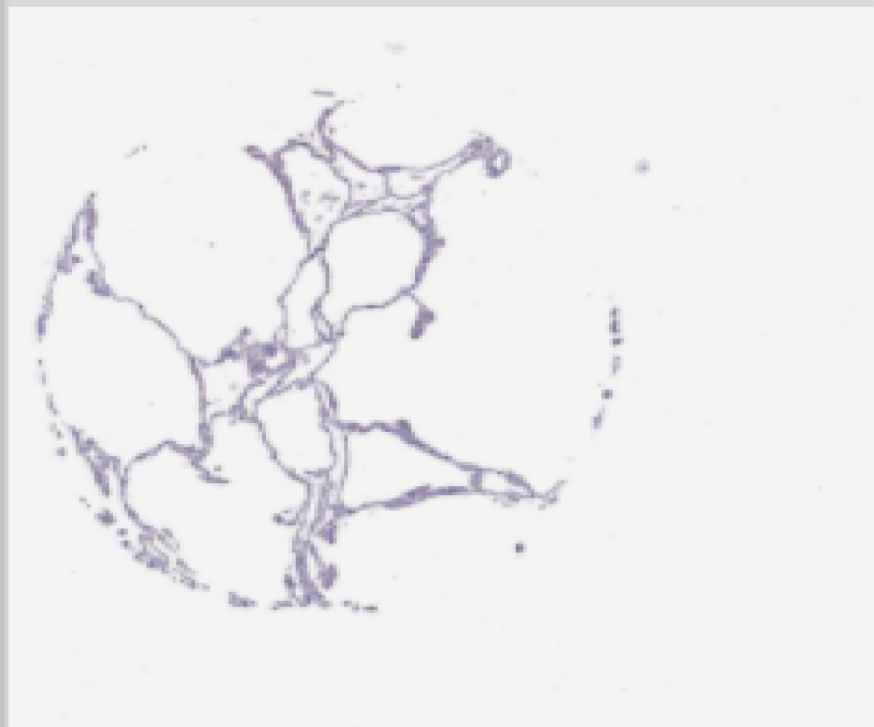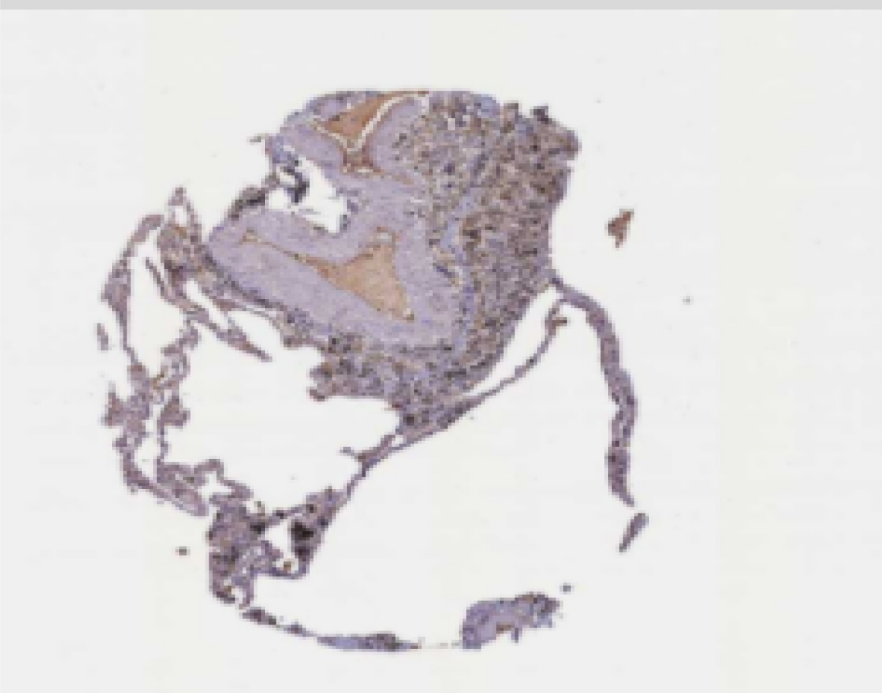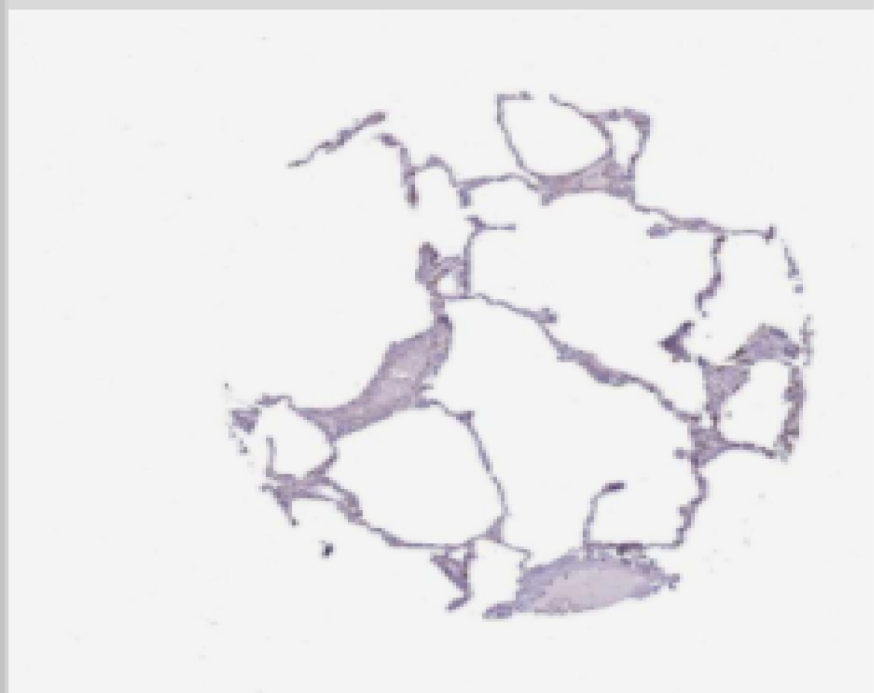

Immunohistochemistry of normal lung tissue tissue shows staining of ACSBG1 .

Supplement: Supplementary 8 — Supplementary Figure 8: immunohistochemistry of normal lung tissues showed staining of ACSBG1. [file 9259297.f8.pdf]
