# Supplementary material for: Comprehensive Analysis of Alteration Landscape and Its Clinical Significance of Mitochondrial Energy Metabolism Pathway-Related Genes in Lung Cancers
Source: Oxid Med Cell Longev. 2021 Dec 20;2021:9259297. doi: 10.1155/2021/9259297 (PMC8713050; doi:10.1155/2021/9259297)
Supplement: Supplementary 3 — Supplementary Figure 3: GSEA enrichment analysis for lung cancer stages I vs. II. [file 9259297.f3.pdf]

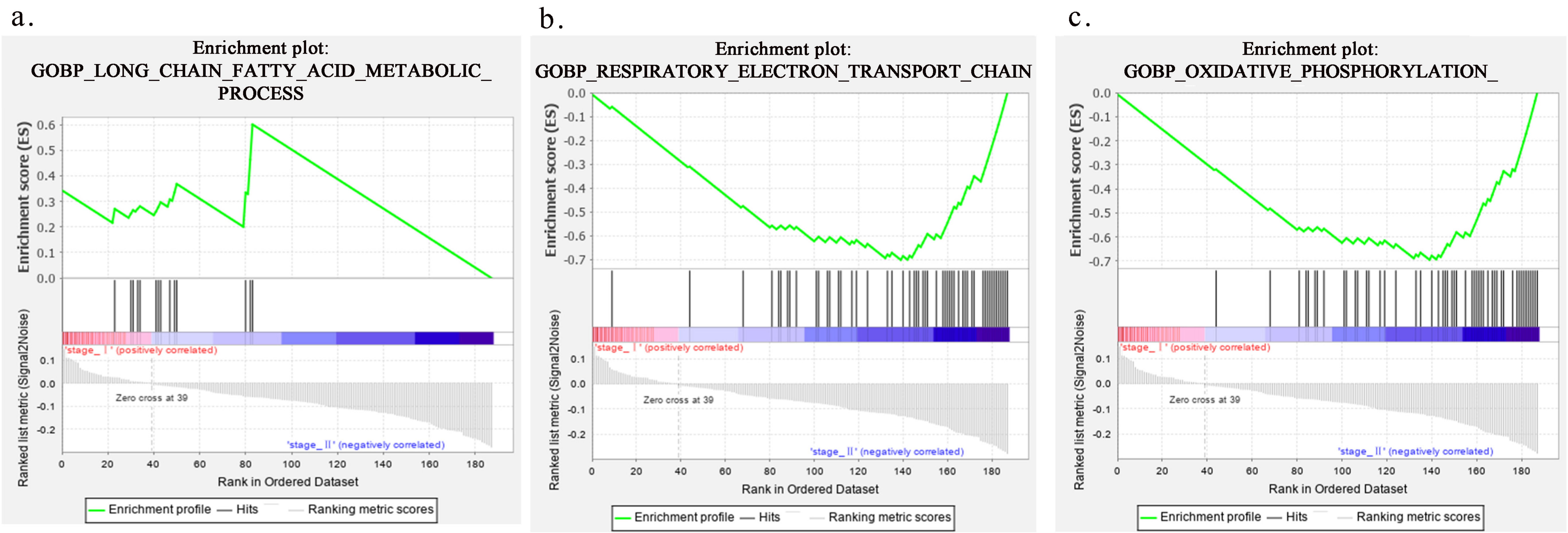

GSEA enrichment analysis for stage I vs.stage II lung cancer.
