# Supplementary material for: Comprehensive Analysis of Alteration Landscape and Its Clinical Significance of Mitochondrial Energy Metabolism Pathway-Related Genes in Lung Cancers
Source: Oxid Med Cell Longev. 2021 Dec 20;2021:9259297. doi: 10.1155/2021/9259297 (PMC8713050; doi:10.1155/2021/9259297)
Supplement: Supplementary 9 — Supplementary Figure 9: the expression of survival-related differentially expressed MMRGs in TCGA and GTEX database of lung cancer. [file 9259297.f9.pdf]

GAPDHS

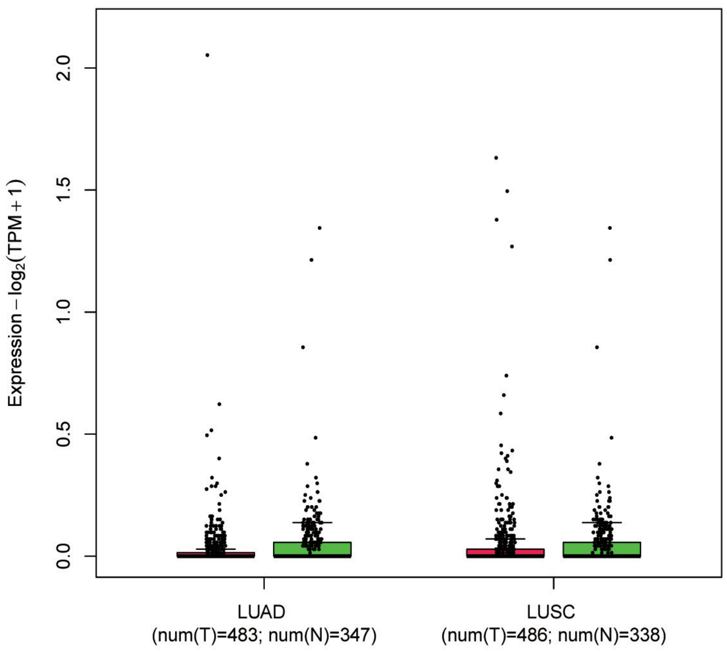

ACSBG1

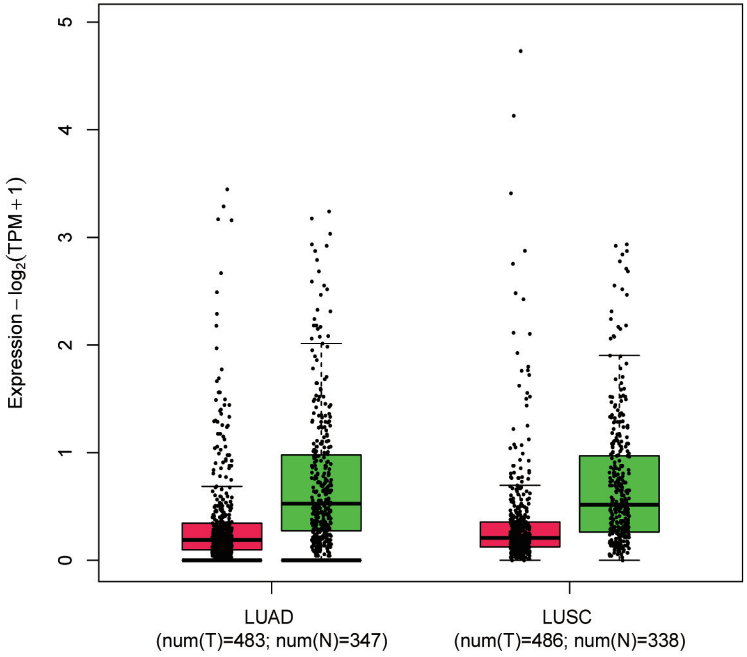

CYP4A11

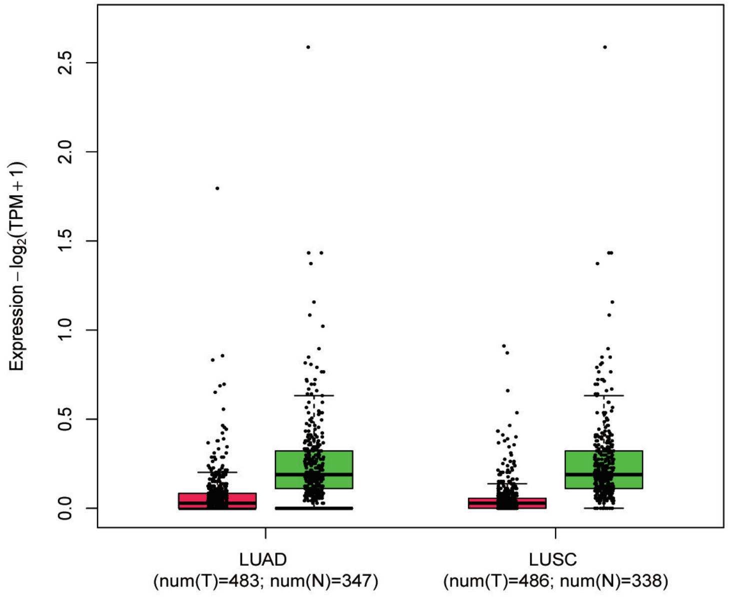

In different types of lung cancer, the expression of three genes in cancer group and normal group (red for cancer group, green for normal group)
